# Strategies used during the cognitive evaluation of older adults with dual sensory impairment: a scoping review

**DOI:** 10.1093/ageing/afae051

**Published:** 2024-03-20

**Authors:** Shirley Dumassais, Margaret Kathleen Pichora-Fuller, Dawn Guthrie, Natalie A Phillips, Marie Savundranayagam, Walter Wittich

**Affiliations:** School of Optometry, Université de Montreal, Montreal, Quebec, H3T 1P1, Canada; Department of Psychology, University of Toronto, Mississauga, Ontario, M5S 3G3, Canada; Department of Kinesiology & Physical Education, Wilfrid Laurier University, Waterloo, Ontario, N2L 3C5, Canada; Department of Health Sciences, Wilfrid Laurier University, Waterloo, Ontario, N2L 3C5, Canada; Department of Psychology/Centre for Research in Human Development, Concordia University, Montreal, Quebec, H4B 1R6, Canada; School of Health Studies, Western University, London, Ontario, N6A 5B9, Canada; School of Optometry, Université de Montreal, Montreal, Quebec, H3T 1P1, Canada; Centre for Interdisciplinary Research in Rehabilitation of Greater Montreal, Montreal, Quebec, H3S 1M9, Canada; Centre de réadaptation Lethbridge-Layton-Mackay du Centre intégré universitaire de santé et de services sociaux du Centre-Ouest-de-l’Île-de-Montréal, Montreal, Quebec, H4B 1T3, Canada

**Keywords:** Cognitive evaluation, Dual sensory impairment, Visual impairment, Hearing impairment, Scoping review, Systematic review, Older people

## Abstract

**Background:**

Dual sensory impairment (DSI), the combination of visual and hearing impairments, is associated with increased risk for age-related cognitive decline and dementia. Administering cognitive tests to individuals with sensory impairment is challenging because most cognitive measures require sufficient hearing and vision. Considering sensory limitations during cognitive test administration is necessary so that the effects of sensory and cognitive abilities on test performance can be differentiated and the validity of test results optimized.

**Objective:**

To review empirical strategies that researchers have employed to accommodate DSI during cognitive testing of older adults.

**Methods:**

Seven databases (*MEDLINE, Embase, Web of Science, CINAHL, PsycINFO, Global Health* and the *Evidence-Based Medicine Reviews* databases) were searched for relevant articles integrating the three concepts of cognitive evaluation, aging, and DSI. Given the inclusion criteria, this scoping review included a total of 67 papers.

**Results:**

Twenty-eight studies reported five categories of strategies for cognitive testing of older adult participants with DSI: the assistance of experts, the modification of standardized test scoring procedures, the use of communication strategies, environmental modifications, and the use of cognitive tests without visual and/or auditory items.

**Conclusions:**

The most used strategy reported in the included studies was drawing on the assistance of team members from related fields during the administration and interpretation of cognitive screening measures. Alternative strategies were rarely employed. Future research is needed to explore the knowledge-to-practice gap between research and current clinical practice, and to develop standardized testing strategies.

## Key Points

- The prevalence of both sensory and cognitive impairments increases with age.
- Commonly used standardized cognitive tests presume sufficient hearing and/or vision for test administration.
- Individuals with sensory impairment may under-perform on cognitive tests, and proactive accommodation is essential.

## Introduction

Age-related sensory and cognitive changes affect the results of behavioural tests administered in the clinical context and how individuals function in daily life [1–3]. Hearing impairment (HI) in mid-life was identified as the largest potentially modifiable risk factor for dementia, while vision impairment (VI) has been associated with greater rates of cognitive dysfunction compared to rates in age-matched healthy individuals [4–9]. Baltes and Lindenberger estimated that vision and hearing account for around 93% of the age-related variability in cognition [10]. Furthermore, hearing and vision may be predictors of cognitive function [11].

Auditory-cognitive and visual-cognitive associations are compounded when people have declines in both senses. Dual sensory impairment (DSI) is the combination of VI and HI, where one sense cannot compensate for the other. Given that both senses are significantly impaired, DSI is a complex condition in which the effects of the sensory-cognitive aging link are intensified [12–14]. Most older adults develop DSI as a result of uncorrectable age-related causes such as age-related macular degeneration and reduced auditory temporal processing [15–17]. Consequently, older adults with DSI are a highly vulnerable population at risk of further sensory as well as cognitive decline. A scoping review investigating the prevalence of DSI in population-based studies revealed a wide range of prevalence rates among adults aged 50 years and above, ranging from 1.6% to 18.2% and higher prevalence of DSI in this age group compared to younger adults [18]. Notably, sex- and gender-related differences have emerged as noteworthy factors in both hearing and vision impairments. HI is more prevalent in males aged 45–85 years old than in age-matched females, while VI is more common in females than in males [19–22].

The strength of the connections between sensory and cognitive declines may be influenced, at least in part, by methodological limitations insofar as cognitive deficits may be over-diagnosed in individuals with sensory impairment due to decreased quality of the sensory inputs when cognitive testing relies on stimuli presented during auditory and visual tasks [6]. Cognitive tests require sufficient hearing and vision for those being tested to understand the instructions and perceive the visual and/or auditory stimuli [23, 24]. However, these tests are commonly administrated in individuals living with HI and/or VI without fully considering that sensory loss may undermine the accuracy of test results. For example, the cognitive effort required by individuals with DSI during cognitive testing is potentially increased because the quality of both the auditory and visual stimuli is reduced and one sense cannot be used to compensate for the other. Such increased effort during perception likely has negative effects on cognitive test performance [25]. When HI is simulated in cognitively healthy adults, test scores on auditory-based cognitive tests (e.g. memory tests) decrease significantly compared to performance in standard conditions [26]. Performance on cognitive tasks that require vision (e.g. the Stroop colour word test) is also significantly reduced in older individuals when visual disorders such as cataracts and decreased colour perception are simulated [27, 28]. Furthermore, VI and HI have each been associated with poorer outcomes on cognitive assessments as illustrated by lower scores on cognitive screening tests that include multiple cognitive domains (e.g. *Mini-Mental State Examination (MMSE)* and *Montreal Cognitive Assessment* (*MoCA)*) [29, 30].

There are currently no evidence-based clinical guidelines for administering existing cognitive tests to older adults with both VI and HI and there are no standardized and validated specialized cognitive tools to test those with DSI. To our knowledge, the literature reports a few cognitive evaluation tools designed for individuals living with DSI who rely on the tactile modality [31–34]. However, these tools have not been integrated into clinical practice and have not yet been validated. The purpose of this scoping review was to explore accommodations for sensory impairment used in research during the cognitive evaluation of older adults with DSI.

## Methods

Given the exploratory nature of this study, a scoping review was the preferred methodological approach. We adhered to the *Joanna Briggs Institute* methodology for scoping reviews [35]. The protocol was developed using the *Preferred Reporting Items for Systematic Review and Meta-Analysis Protocols (PRISMA-P)* statement and reporting of this review was done using the *PRISMA Extension for Scoping Reviews (PRISMA-ScR)* [36, 37].

### Eligibility criteria

Studies focusing on older adults with DSI were eligible for inclusion because of that population’s increased risk of cognitive decline [38]. Participants in eligible studies had to have both HI and VI as identified using behavioural tests or self-reports, regardless of the nature and aetiology of their sensory losses. Participants were not required to be diagnosed with, or have suspected cognitive decline, as this review addressed measures used to evaluate cognitive performance. Studies that did not focus on cognitive measures and related strategies were excluded. Any study considering cognitive measures in older adults with any sensory impairment was included in the early stages of the scoping review, but at the stage of full-text review, the inclusion criteria were narrowed to only include studies including participants with DSI, who were at least 65 years old. The final study eligibility criteria are outlined in Table 1.

**Table 1:** Scoping review inclusion and exclusion criteria

**Abstract and title screening**	
Include	Exclude
Studies that consider the cognitive status of the individuals with hearing and/or visual impairment with standardized cognitive tests. This includes studies that assess/measure cognitive domains such as short & long-term memory, executive function, processing speed, language, verbal skills, etc. Studies that consider participants with sensory loss (visual impairment, hearing impairment, dual sensory impairment). It is not mandatory that all participants have hearing and/or visual impairment. Studies that explore modifications to cognitive screening tests for sensory loss. Studies that include older adults in their participants (65+). It is not mandatory that all participants are older adults.	Studies in which the cognitive function of individuals with hearing and/or visual impairment is not considered. Studies in which participants do not have hearing and/or visual impairment. Studies in which participants do not include individuals aged 65 and older. Studies which focus only on psychological/mental health aspects (i.e. depression, anxiety) but not cognition. Studies in which dual sensory impairment (if applicable) is about senses other than hearing and vision. Animal model studies. Editorials, comments, conference publications, academic thesis/dissertations, books, or letters.
**Full text review**	
Study sample must include at least one participant with dual sensory impairment.	Articles that are not written in English, French, Portuguese, or German.

### Information sources

Six databases *(MEDLINE, Embase, Web of Science, CINAHL, PsycINFO, and Global Health)* were searched for articles in accordance with *PRISMA*. Furthermore, all eight databases from the *Evidence-Based Medicine Reviews* database were searched.

### Search

The concepts and database search strategies were developed with the collaboration and expertise of an experienced librarian at the *Université de Montréal*’s School of Optometry. Search terms and keywords were explored through initial searches in the PubMed database. Relevant keywords were searched for synonyms, broader and narrower terms, as well as alternate spellings and were adapted for searches in the different selected databases.

Three main concepts were explored: (A) cognitive evaluation; (B) DSI; and (C) older adults. Cognitive evaluation refers to any measure or tool to evaluate cognitive domains (e.g. memory, language, abstraction, executive function, attention) either for cognitive screening or neuropsychological assessment. DSI was defined as a condition that combines both HI and VI [12]. Impairments could be defined using self-report, questionnaires, or behavioural measures (e.g. tests of visual acuity, contrast sensitivity, pure-tone, or speech-in-noise thresholds). Older adults were defined as individuals 65 years of age or older. No studies were excluded based on the sex and gender of their participants, their spoken languages, communication preferences, or comorbid health conditions.

### Selection of sources of evidence

This review explored peer-reviewed studies that adapted cognitive tools or evaluated adapted tools, their administration, their scoring or their interpretation to accommodate the comprehension of test instructions or the perception of test stimuli by older individuals with DSI. Selected studies included any research design providing empirical data that were collected in any setting. Searches were not limited in publication time or location but were limited to publications in the French, English, Portuguese and German languages. Editorials, commentaries, conference publications, theses and dissertations, books or letters were excluded. Searches were performed in May 2022. An example of the search strategy performed in the *MEDLINE* database is provided in Appendix A.

The articles resulting from database searches were exported to the EndNote X9 software (Clarivate Analytics, PA, USA) and subsequently to the Covidence software (Veritas Health Innovation, Melbourne, Australia) where article duplicates were removed [39, 40]. Within Covidence, each title, abstract and full article was reviewed by two independent reviewers using the criteria presented in Table 1. Any conflict in decisions was reviewed and resolved by a senior reviewer.

### Data charting and synthesis of results

Articles that met the inclusion criteria had their data extracted into a *Microsoft Excel* pre-designed spreadsheet. Data were synthesized in a table and included qualitative and quantitative information such as study design and setting, sample characteristics such as sex/gender and age, type of vision and hearing assessments, cognitive tests used by researchers/professionals, administration strategies and psychometric test properties (if reported), as well as study results and main points.

## Results

### Selection of evidence sources

**Figure 1:**
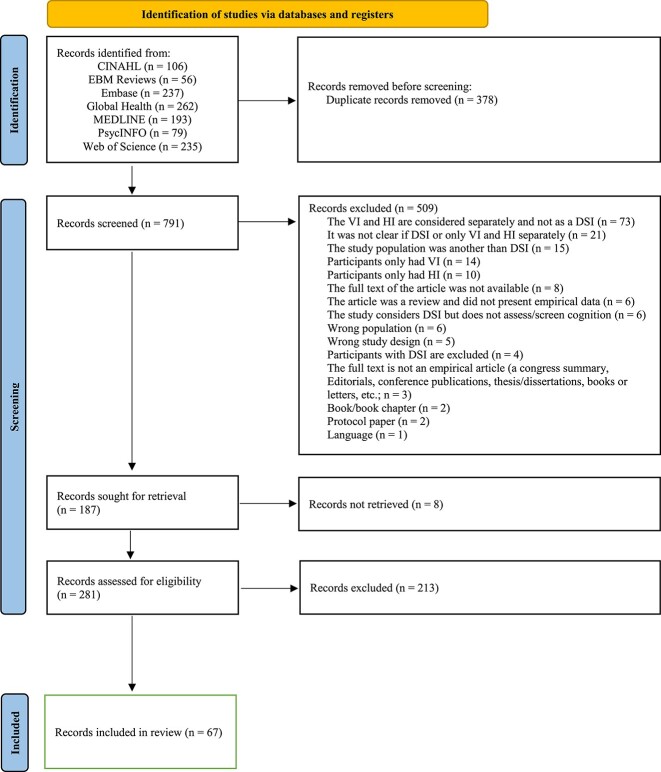
PRISMA Flow Diagram.

The database searches yielded 1,169 publications. After removing duplicates, the titles and abstracts of 791 articles were screened and 281 studies were deemed eligible to be reviewed comprehensively. At the stage of full-text review, 213 studies were excluded for reasons such as the absence of cognitive evaluation, or sample population characteristics. A final total of 67 papers exploring the cognitive evaluation of older adults living with actual, non-simulated DSI were included (see PRISMA flow chart in Figure 1).

### Characteristics of evidence sources

#### Study information

Included studies dated back to 1998, up to May 2022. Of the 61 studies that reported their setting, 12 were conducted in long-term care homes, ten were conducted in clinics or hospitals and one in a research laboratory, while others were conducted in various community settings. Most articles utilized a cross-sectional design (*n* = 37) while 30 were longitudinal studies.

#### Study participants’ characteristics

In 42 studies, the age range of the participants was not specified. In 19 studies, participants were from different age categories, including young-old individuals (aged 65–74 years), middle-old individuals (aged 75–84 years), and old-old individuals (over 85 years). An additional five studies primarily recruited young-old and middle-old participants, while only one study specifically focused on middle-old and old-old individuals. Among the reviewed studies, 53 used terminologies interchanging sex and gender; 12 studies exclusively referred to the sex of their participants, utilizing terms such as male and female; two studies referred to the gender of participants. Fifty studies provided the ratio of the sex and/or gender of their overall participant sample. In those, 25 studies had an equal distribution of both sexes/genders; the 25 other studies had a biased representation of females/women with one study exclusively including females/women as participants. For studies that only presented data on sensory subgroups of their sample (*n* = 12), nine studies indicated a balanced distribution of males and females among individuals with DSI; two studies displayed a bias toward males; one study exhibited a bias toward females. Notably, five studies did not provide any data pertaining to the sex or gender distribution within the DSI subgroup.

#### Sensory measures

All but two studies performed vision and hearing tests to identify sensory loss as part of their research protocols. However, Appendix B illustrates significant variability in the methods used to ascertain sensory loss, highlighting the lack of a standardized assessment method for sensory impairment prior to cognitive evaluations. In 30 studies, behavioural vision tests (e.g. the *Snellen* chart, the *Early Treatment Diabetic Retinopathy Study*) were used, 28 studies used self-report screening tools of visual function; seven studies included both self-report and behavioural measures of vision. As for measures of hearing, 24 studies employed behavioural measures (e.g. pure-tone audiometry); 33 studies used self-report measures; eight studies used both self-report and behavioural tests.

#### Cognitive measures

A total of 23 different cognitive tools were reported across the included studies, 19 screening tools and 4 diagnostic tools. The *MMSE* was the most commonly used (*n* = 27) [41]. Thirteen studies evaluated specific cognitive domains with the use of cognitive subtests; for example, the *Digit Span Backwards subtest the revised Wechsler Adult Intelligence Scale WAIS–R* was used to measure working memory and animal naming to measure verbal fluency [42]. The *Cognitive Performance Scale (CPS)* was used in 11 studies [43]. Four studies used the *Telephone Interview for Cognitive Status (TICS)* [44]. Dementia diagnosis was reported in four studies. The *MoCA* [45] and the *Clinical Dementia Rating* [46] were each used by three studies. Other reported measures included neuropsychological test batteries to establish the presence or absence of dementia (*n* = 2), and self-reported perception of cognitive function (*n* = 2).

Only one of the studies used a measure that accommodated for DSI. This cognitive tool consisted of a tactile test battery that did not require participants to have vision or hearing [31]. In this study, task instructions were provided by trained consultants using tactile sign language, spoken language, by use of written texts or other means adapted to the participants’ communication requirements. The cognitive tasks included tactile versions of a spatial memory test, a clock reading test, a tactile form board test and naming tests. Seven studies used tests that inherently accommodate for visual impairment. These included cognitive evaluations conducted over the phone, without the presentation of visual items, or tests where the visually presented items were not completed by participants and the scoring was modified accordingly [31, 44, 47]. However, none of the studies used a test that inherently accommodated for hearing impairment, such as by using tests that are vision-based only or pocket-talkers [48–50].

#### Strategies employed during the administration of cognitive tests

All identified cognitive evaluation tools and strategies to administer cognitive tests are provided in Appendix B. Of the 67 included articles (full bibliography available in Appendix C), only 28 studies made use of strategies during the administration or the interpretation of a cognitive measure. Five categories of strategies were identified: (A) the assistance of experts (18 studies) (B) the modification of standardized tests’ scoring procedures (four studies), (C) the use of adaptive communication strategies (four studies) (D) environmental modifications (two studies) and (E) the use of cognitive tests without visual and/or auditory items (six studies). Details of these strategies are included in Appendix B.

Six studies employed cognitive tests that, by design, did not include visual items, such as the *Telephone Interview for Cognitive Status* (TICS), and/or auditory items such as tactile tests [13, 31, 44, 51–53]. Another reported strategy was the modification of established standardized tests’ scoring procedures. For instance, the *MoCA* and the *MMSE* in their blind versions [29, 54–56] were respectively administered in two articles. In these studies, test total scores were modified by not counting the points awarded for visually presented items. Communication strategies (e.g. use of assistive devices, adapted speech and requesting feedback during the conversation) were also employed to accommodate individuals with DSI in four studies [31, 51, 57, 58]. Only two studies ensured a quiet environment to optimize speech perception [51, 58].

### Strategies for the interpretation of cognitive tests:

In 19 studies [3, 31, 58–72], testing was conducted with the assistance of team members (e.g. research assistants, nurses and/or social workers) trained on various aspects of sensory and cognitive aging. In some studies, deafblind/DSI specialists, trained research assistants or nurses administered tests to individuals with DSI and served as consultants during the sessions. In other studies, clinical psychologists, geriatricians, and geriatric psychiatrists were actively involved in the consensus process to develop the clinical profile of the study participants. Although these professionals did not directly accommodate for sensory impairment, they were able to use specialist knowledge to assist the research team in contextualizing the sensory impairment when interpreting cognitive test scores [59, 63, 65].

#### Psychometric properties

None of the 28 studies that accommodated cognitive test administration for older adults with DSI conducted validity analyses to ensure that their strategies did not alter the way in which the cognitive tools measure cognitive function. One article established the consistency of the cognitive measure they used across test administrators by reporting an inter-rater reliability of 90%, demonstrating very good agreement across test administrators [66]. Sensitivity and specificity can be determined through comprehensive statistical analyses, as demonstrated in the adaptation of the Montreal Cognitive Assessment (MoCA) for individuals with visual impairment [47]. Alternatively, these metrics can be derived from comparative assessments, as highlighted in the study by Utoomprurkporn et al. (2021) [73] which involved evaluating a cognitive tool tailored for hearing loss among older individuals with diverse levels of cognitive function and hearing impairment.

## Discussion

The present review aimed to identify the strategies used during the cognitive evaluation of older adults with DSI. Notably, we discovered a lack of such accommodations and consideration of the sensory profile of participants. Most studies that did assess vision and hearing used self-report measures. However, combining self-report with behavioural tests should give a more complete assessment of the sensory abilities of older adults [74]. Cognitive evaluation without behavioural sensory assessments is highly problematic because cognitive scores may be lower due to sensory limitations that are not revealed using self-report measures and that the tester is not aware of [6]. There are currently no validation studies of appropriate cognitive measures for people with DSI. There is a need to develop tools so that the cognitive abilities of older individuals living with DSI can be assessed independent of sensory limitations.

### Effect of clinical setting on cognitive evaluation

The included studies demonstrate a paucity of solutions across the five identified categories. The majority of studies that adapted their protocol were conducted in long-term care homes, an environment with a high prevalence of residents with combined sensory and cognitive impairments [3]. In this environment, it is especially important to accommodate sensory impairments when testing cognition. Considering sensory impairments when interpreting cognitive test scores will provide more accurate assessments that may contribute to better person-centered approaches to care. Cognitive evaluations encompass both screening for the purpose of referral, and assessment for diagnostic purposes. The researchers working in long-term care homes tended to use the assistance of trained experts to conduct and interpret cognitive testing in older adults with DSI. This approach likely increases how informative assessments are because the expertise of specialists can substantiate the interpretation of tests results and provide person-centered context. However, depending on the institutional setting, such experts may not be involved in screening and there may be delays in referral for assessments. Until tests validated for use with people living with DSI are developed, better inter-professional collaboration and training in how to accommodate sensory impairment during testing could reduce delays and reliance on costly and time-consuming resources. Client-centered strategies such as the provision of visual and/or hearing aids as well as other assistive technologies and strategies could benefit those with DSI who are at risk for, or living with cognitive decline.

### Challenges in standardized testing for concurrent vision and hearing impairment

Very few articles included in this review employed communication strategies or environmental modifications for people with DSI. Only four studies used standardized cognitive tests where the visually presented items were removed, and the scoring was adjusted accordingly. The *MoCA-Blind,* without visually presented items*,* has been validated against its full version with the visually presented items. Specifically, withdrawing the visual items from the test and adjusting scoring generated excellent test specificity (98%) and adequate sensitivity (63%) in individuals with mild cognitive impairment, with the caveat that this approach omits screening of some cognitive functions when visual test items are removed [47]. The blind version of the *MMSE* uses age-dependent cut-off values, has been validated, and reports excellent sensitivity (91–100%) and specificity (80–100%) [75]. Such assessments do not require clients to visually perceive test items and can be administered over the phone. The obstacle with these measures is that they still do not accommodate for hearing impairment, especially when such measures are administered remotely. A version of the *MoCA* that is presented visually (no hearing required) has been explored but yielded less sensitivity than the MoCA-Blind [24].

Researchers often make statistical choices to manage population-based data that include individuals with sensory impairment. For example, in this scoping review, we identified a study that managed the effect of sensory impairment on cognitive outcomes by analysing the data with and without participants who had mild cognitive impairment or severe cognitive dysfunction. The authors made this choice because they were unsure if severe cognitive impairment would interfere with the participants’ ability to follow the instructions required to complete the vision and hearing measures [68, 70]. However, it would be better to test for, and accommodate, sensory impairments in advance of cognitive testing. The tactile test battery proposed by Bruhn and Dammeyer is an innovative step toward the validation of a measure for older adults living with DSI [31]. The psychometric properties of such adapted cognitive measures will need to be revisited for administration in older adults with DSI. Unfortunately, only one article reported reliability data, and none provided validity data. An interprofessional team will likely need to collaborate on developing and validating appropriate cognitive tests for people living with DSI, including experts in neuropsychology and sensory impairment. However, there is a potential risk that these adapted tools will be considered more cumbersome as they may require additional testing material, training, and time for practitioners.

## Limitations

DSI only recently started receiving increased scientific attention given the increase in its prevalence [76]. It may be too early to expect progress in research or clinical practice to develop and implement novel strategies given that rigorous standardization and validation is a lengthy process. It is likely that existing adaptive strategies specific to the administration of cognitive tests in individuals with single sensory impairment have been missed because our review focused on DSI. Ignoring such strategies prevents cross-comparisons with those that may be suitable with clients living with DSI. Furthermore, it would be relevant to explore the effect of degree of sensory impairment, as well as the effectiveness of using assistive technologies and environmental modifications for individuals with sensory impairments of all ages to compare across age groups.

Future studies should also explore how sex- and gender-related differences may influence sensory-cognitive links, best practices for accommodating sensory loss during cognitive testing, and for subsequent care planning. More generally, future studies should include participants with a diverse range of characteristics, including but not limited to race, education, communication modalities, language, and the presence of comorbidities. By actively addressing these factors, researchers can foster a more representative and comprehensive understanding of various populations, thereby promoting the validity and applicability of study findings.

Failure to optimize cognitive testing for individuals with sensory impairment may lead to negative consequences, such as the misinterpretation of results due to a lack of consideration of their sensory context [76, 77]. There is a risk of reduced test integrity by modifying the scoring techniques, the individual test items or the use of non-standardized methods of administration [24, 76]. Misinterpretations of cognitive evaluations may have serious consequences for research and care for older adults with DSI.

## Supplementary Material

aa-23-1214-File002_afae051

## References

[1] Abrahamson K , Clark D, Perkins A, Arling G. Does cognitive impairment influence quality of life among nursing home residents? Gerontologist 2012; 52: 632–40. 22230491 10.1093/geront/gnr137 PMC3463417

[2] Bouscaren N , YildizH, DartoisL, VercambreMN, Boutron-RuaultMC. Decline in instrumental activities of daily living over 4-year: the association with hearing, visual and dual sensory impairments among non-institutionalized women. J Nutr Health Aging2019; 23: 687–93.31560024 10.1007/s12603-019-1231-9

[3] Guthrie DM , Davidson JGS, Williams N et al. Combined impairments in vision, hearing and cognition are associated with greater levels of functional and communication difficulties than cognitive impairment alone: analysis of interRAI data for home care and long-term care recipients in Ontario. 2018; 13: e0192971. 10.1371/journal.pone.0192971 PMC5814012 29447253

[4] Livingston G , Huntley J, Sommerlad A et al. Dementia prevention, intervention, and care: 2020 report of the lancet commission. Lancet (London, England) 2020; 396: 413–46. 32738937 10.1016/S0140-6736(20)30367-6 PMC7392084

[5] Nagarajan N , Assi L, Varadaraj V et al. Vision impairment and cognitive decline among older adults: a systematic review. BMJ Open 2022; 12: e047929. 10.1136/bmjopen-2020-047929 PMC8739068 34992100

[6] Uchida Y , Saigura S, Nishita Y et al. Age-related hearing loss and cognitive decline — the potential mechanisms linking the two. Auris Nasus Larynx 2019; 46: 1–9. 30177417 10.1016/j.anl.2018.08.010

[7] Füllgrabe C . On the possible overestimation of cognitive decline: the impact of age-related hearing loss on cognitive-test performance. Front Neurosci 2020; 14: 454. 32581666 10.3389/fnins.2020.00454 PMC7296091

[8] Ehrlich JR , Goldstein J, Swenor BK, Whitson H, Langa KM, Veliz P. Addition of vision impairment to a life-course model of potentially modifiable dementia risk factors in the US. JAMA Neurol 2022; 79: 623–6. 35467745 10.1001/jamaneurol.2022.0723 PMC9039828

[9] Lin FR , Pike JR, Albert MS et al. Hearing intervention versus health education control to reduce cognitive decline in older adults with hearing loss in the USA (ACHIEVE): a multicentre, randomised controlled trial. The Lancet (London, England) 2023; 402: 786–97. 37478886 10.1016/S0140-6736(23)01406-X PMC10529382

[10] Baltes PB , Lindenberger U. Emergence of a powerful connection between sensory and cognitive functions across the adult life span: a new window to the study of cognitive aging? Psychol Aging 1997; 12: 12–21. 9100264 10.1037//0882-7974.12.1.12

[11] Phillips NA , Isler L, Kabir R et al. Hearing and visual acuity predict cognitive function in adults aged 45–85 years: findings from the baseline wave of the Canadian longitudinal study on aging (CLSA). Psychol Aging 2022; 37: 891–912. 36355655 10.1037/pag0000716

[12] Nordic Centre for Welfare and Social Issues . Nordic Definition of Deafblindness. Accessed from: http://www.nordicwelfare.org/wp-content/uploads/2018/03/nordic-definition-of-deafblindness.pdf

[13] Maharani A , Dawes P, Nazroo J et al. Associations between self-reported sensory impairment and risk of cognitive decline and impairment in the health and retirement study cohort. J Gerontol B Psychol Sci Soc Sci 2020; 75: 1230–42. 30977823 10.1093/geronb/gbz043

[14] Lin MY , Gutierrez PR, Stone KL et al. Vision impairment and combined vision and hearing impairment predict cognitive and functional decline in older women. J Am Geriatr Soc 2004; 52: 1996–2002. 15571533 10.1111/j.1532-5415.2004.52554.x

[15] World Health Organization . Vision Impairment and Blindness. Accessed from: https://www.who.int/news-room/fact-sheets/detail/blindness-and-visual-impairment.

[16] World Health Organization . Deafness and Hearing Loss. Accessed from: https://www.who.int/news-room/fact-sheets/detail/deafness-and-hearing-loss

[17] Wittich W , Simcock P. Aging and combined vision and hearing loss. The Routledge Handbook of Visual Impairment. Routledge Taylor & Francis Group, 2019, 438–56.

[18] Bright T , Ramke J, Zhang JH et al. Prevalence and Impact of Combined Vision and Hearing (Dual Sensory) Impairment: A Scoping Review. Robinson J (ed.). PLOS global public health 2023; 3: e0001905. 10.1371/journal.pgph.0001905 PMC10187940 37192147

[19] Hayward LM , Burden ML, Burden AC et al. What is the prevalence of visual impairment in the general and diabetic populations: are there ethnic and gender differences? Diabet Med 2002; 19: 27–34. 10.1046/j.0742-3071.2001.00603.x 11869300

[20] Mick P , Hämäläinen A, Kolisang L et al. The prevalence of hearing and vision loss in older Canadians: an analysis of the Canadian longitudinal study on aging. Canadian Journal on Aging / La Revue canadienne du vieillissement 2020; 40: 1–22. 32546290 10.1017/S0714980820000070

[21] Aljied R , Aubin M-J, Buhrmann R, Sabeti S, Freeman EE. Prevalence and determinants of visual impairment in Canada: cross-sectional data from the Canadian longitudinal study on aging. Can J Ophthalmol 2018; 53: 291–7. 29784168 10.1016/j.jcjo.2018.01.027

[22] Hämäläinen A , Pichora-Fuller MK, Wittich W, Phillips NA, Mick P. Self-report measures of hearing and vision in older adults participating in the Canadian longitudinal study of aging are explained by Behavioral sensory measures, demographic, and social factors. Ear Hear 2021; 42: 814–31. 33741763 10.1097/AUD.0000000000000992

[23] Brown J . The use and misuse of short cognitive tests in the diagnosis of dementia. J Neurol Neurosurg Psychiatry 2015; 86: 680–5. 25411547 10.1136/jnnp-2014-309086

[24] Al-Yawer F , Pichora-Fuller MK, Phillips NA. The Montreal cognitive assessment after omission of hearing-dependent subtests: psychometrics and clinical recommendations. J Am Geriatr Soc 2019; 67: 1689–94. 31018015 10.1111/jgs.15940

[25] Pichora-Fuller MK , Kramer SE, Eckert MA et al. Hearing impairment and cognitive energy: the framework for understanding effortful listening (FUEL). Ear Hear 2016; 37: 5S–27. 27355771 10.1097/AUD.0000000000000312

[26] Jorgensen LE , Palmer CV, Pratt S, Erickson KI, Moncrieff D. The effect of decreased audibility on MMSE performance: a measure commonly used for diagnosing dementia. J Am Acad Audiol 2016; 27: 311–23. 27115241 10.3766/jaaa.15006

[27] Wood J , Chaparro A, Anstey K et al. Simulated visual impairment leads to cognitive slowing in older adults. Optometry and vision science : official publication of the American Academy of Optometry 2010; 87: 1037–43. 21037492 10.1097/OPX.0b013e3181fe64d7

[28] Ben-David BM , Schneider BA. A sensory origin for color-word Stroop effects in aging: simulating age-related changes in color-vision mimics age-related changes in Stroop. Aging, Neuropsychology, and Cognition 2010; 17: 730–46. 10.1080/13825585.2010.510553 21058053

[29] Dupuis K , Pichora-Fuller MK, Chasteen AL, Marchuk V, Singh G, Smith SL. Effects of hearing and vision impairments on the Montreal cognitive assessment. Neuropsychol Dev Cogn B Aging Neuropsychol Cogn 2015; 22: 413–37. 25325767 10.1080/13825585.2014.968084

[30] Lim MYL , Loo JHY. Screening an elderly hearing impaired population for mild cognitive impairment using mini-mental state examination (MMSE) and Montreal cognitive assessment (MoCA). Int J Geriatr Psychiatry 2018; 33: 972–9. 29575215 10.1002/gps.4880

[31] Bruhn P , Dammeyer J. Assessment of dementia in individuals with dual sensory loss: application of a tactile test battery. Dementia and geriatric cognitive disorders extra 2018; 8: 12–22. 29515619 10.1159/000486092 PMC5836148

[32] Arnold P , Heiron K. Tactile memory of deaf-blind adults on four tasks. Scand J Psychol 2002; 43: 73–9. 11885762 10.1111/1467-9450.00270

[33] Janssen MJ , Nota S, Eling PATM, Ruijssenaars WAJJM. The advantage of encoding tactile information for a woman with congenital deaf-blindness. Journal of Visual Impairment & Blindness 2007; 101: 653–7.

[34] Papagno C , Minniti G, Mattavelli GC, Mantovan L, Cecchetto C. Tactile short-term memory in sensory-deprived individuals. Exp Brain Res 2017; 235: 471–80. 27785548 10.1007/s00221-016-4808-0

[35] Peters MDJ , Godfrey CM, Khalil H, McInerney P, Parker D, Soares CB. Guidance for conducting systematic scoping reviews. International journal of evidence-based healthcare. Int J Evid Based Healthc 2015; 13: 141–6. 26134548 10.1097/XEB.0000000000000050

[36] Tricco AC , Lillie E, Zarin W et al. PRISMA extension for scoping reviews (PRISMA-ScR): checklist and explanation. Ann Intern Med 2018; 169: 467–73. 30178033 10.7326/M18-0850

[37] Moher D , Shamseer L, Clarke M et al. Preferred reporting items for systematic review and meta-analysis protocols (PRISMA-P) 2015 statement. Syst Rev 2015; 4: 1–9. 25554246 10.1186/2046-4053-4-1 PMC4320440

[38] Deary IJ , Corley J, Gow AJ et al. Age-associated cognitive decline. Br Med Bull 2009; 92: 135–52. 19776035 10.1093/bmb/ldp033

[39] The EndNote Team . EndNote. 2013.

[40] Veritas Health Innovation . Covidence systematic review software. In.

[41] Folstein MF , Folstein SE, Mchugh R. “Mini-mental state”. A practical method for grading the cognitive state of patients for the clinician. J Psychiatr Res 1975; 12: 189–98. 1202204 10.1016/0022-3956(75)90026-6

[42] Wechsler D . Wechsler adult intelligence scale. Arch Clin Neuropsychol 1955.

[43] Morris JN , Fries BE, Mehr DR et al. MDS cognitive performance scale®. J Gerontol 1994; 49: M174–82. 8014392 10.1093/geronj/49.4.m174

[44] Brandt J , Folstein MF. The telephone interview for cognitive status. Neuropsychiatry, Neuropsychology, and Behavioural Neurology 1988; 1: 111–7.

[45] Nasreddine ZS , Phillips NA, Bedirian V et al. The Montreal cognitive assessment, MoCA: a brief screening tool for mild cognitive impairment. J Am Geriatr Soc 2005; 53: 695–9. 15817019 10.1111/j.1532-5415.2005.53221.x

[46] Morris JC . The clinical dementia rating (CDR): current version and scoring rules. Neurology 1993; 43: 2412–4. 10.1212/wnl.43.11.2412-a 8232972

[47] Wittich W , Phillips N, Nasreddine ZS, Chertkow H. Sensitivity and specificity of the Montreal cognitive assessment modified for individuals who are visually impaired. Journal of Visual Impairment & Blindness 2010; 104: 360–8.

[48] Shen J , Sherman M, Souza PE. Test administration methods and cognitive test scores in older adults with hearing loss. Gerontology 2021; 66: 24–32. 10.1159/000500777 PMC6930984 31242497

[49] Lin VYW , Chung J, Callahan BL et al. Development of cognitive screening test for the severely hearing impaired: hearing-impaired MoCA: development of hearing-impaired MoCA. Laryngoscope 2017; 127: S4–11. 28409842 10.1002/lary.26590

[50] Saunders GH , Odgear I, Cosgrove A, Frederick MT. Impact of hearing loss and amplification on performance on a cognitive screening test. J Am Acad Audiol 2018; 29: 648–55. 29988012 10.3766/jaaa.17044

[51] Heyl V , Wahl H-W. Managing daily life with age-related sensory loss: cognitive resources gain in importance. Psychol Aging 2012; 27: 510–21. 22059715 10.1037/a0025471

[52] Rong H , Lai X, Jing R, Wang X, Fang H, Mahmoudi E. Association of Sensory Impairments with cognitive decline and depression among older adults in China. JAMA Netw Open 2020; 3: e2014186. 32990739 10.1001/jamanetworkopen.2020.14186 PMC7525357

[53] Zhao X , Zhou Y, Wei K et al. Associations of sensory impairment and cognitive function in middle-aged and older Chinese population: the China health and retirement longitudinal study. J Glob Health 2021; 11: 08008. 34956639 10.7189/jogh.11.08008 PMC8684796

[54] Hong T , Mitchell P, Burlutsky G, Liew G, Wang JJ. Visual impairment, hearing loss and cognitive function in an older population: longitudinal findings from the Blue Mountains eye study. PloS One 2016; 11: e0147646. 26808979 10.1371/journal.pone.0147646 PMC4726694

[55] Mudie LI , Varadaraj V, Gajwani P et al. Dual sensory impairment: the association between glaucomatous vision loss and hearing impairment and function. PloS One 2018; 13: e0199889. 29979753 10.1371/journal.pone.0199889 PMC6034827

[56] Alfaro AU , Guthrie DM, Phillips NA et al. Detection of vision and /or hearing loss using the interRAI community health assessment aligns well with common behavioral vision/hearing measurements. PloS One 2019; 14: e0223123. 31581243 10.1371/journal.pone.0223123 PMC6776414

[57] de la Fuente J , Hjelmborg J, Wod M et al. Longitudinal associations of sensory and cognitive functioning: a structural equation Modeling approach. J Gerontol B Psychol Sci Soc Sci 2019; 74: 1308–16. 30521005 10.1093/geronb/gby147

[58] Wahl H-W , Heyl V, Drapaniotis PM et al. Severe vision and hearing impairment and successful aging: a multidimensional view. Gerontologist 2013; 53: 950–62. 23471603 10.1093/geront/gnt013

[59] Byeon G , Oh GH, Jhoo JH et al. Dual sensory impairment and cognitive impairment in the Korean longitudinal elderly cohort. Neurology 2021; 96: e2284–95. 33827964 10.1212/WNL.0000000000011845

[60] Dammeyer J . Interaction of dual sensory loss, cognitive function, and communication in people who are congenially deaf-blind. Journal of Visual Impairment & Blindness 2010; 104: 719–25.

[61] Guthrie DM , Declercq A, Finne-Soveri H, Fries BE, Hirdes JP. The health and well-being of older adults with dual sensory impairment (DSI) in four countries. PloS One 2016; 11: e0155073. 27148963 10.1371/journal.pone.0155073 PMC4858206

[62] Guthrie DM , Thériault ÉR, Davidson JGS. Self-rated health, cognition, and dual sensory impairment are important predictors of depression among home care clients in Ontario. Home Health Care Manag Pract 2016; 28: 35–43.

[63] Hwang PH Jr , Longstreth WT Jr, Brenowitz WD et al. Dual sensory impairment in older adults and risk of dementia from the GEM study. Alzheimer’s & dementia (Amsterdam, Netherlands) 2020; 12: e12054. 10.1002/dad2.12054 PMC7340796 32671180

[64] Mitoku K , Masaki N, Ogata Y, Okamoto K. Vision and hearing impairments, cognitive impairment and mortality among long-term care recipients: a population-based cohort study. BMC Geriatr 2016; 16: 1–7. 27233777 10.1186/s12877-016-0286-2 PMC4884419

[65] Pabst A , Bär J, Röhr S et al. Do self-reported hearing and visual impairments predict longitudinal dementia in older adults? J Am Geriatr Soc 2021; 69: 1519–28. 33734430 10.1111/jgs.17074

[66] Petrovsky DV , Sefcik JS, Hanlon AL, Lozano AJ, Cacchione PZ. Social engagement, cognition, depression and co-morbidity in sensory impaired nursing home residents. Res Gerontol Nurs 2019; 12: 217–26. 31283831 10.3928/19404921-20190627-01 PMC6756972

[67] Yamada Y , Denkinger MD, Onder G et al. Dual sensory impairment and cognitive decline: the results from the Shelter study. J Gerontol A Biol Sci Med Sci 2016; 71: 117–23. 25869524 10.1093/gerona/glv036

[68] Yamada Y , Vlachova M, Richter T et al. Prevalence and correlates of hearing and visual impairments in European nursing homes: results from the SHELTER study. J Am Med Dir Assoc 2014; 15: 738–43. 24984787 10.1016/j.jamda.2014.05.012

[69] Guthrie DM , Williams N, Campos J et al. A newly identified impairment in both vision and hearing increases the risk of deterioration in both communication and cognitive performance. Canadian Journal on Aging / La Revue canadienne du vieillissement 2022; 41: 363–76. 35859361 10.1017/S0714980821000313

[70] Hwang PH , Longstreth WT, Thielke SM et al. Longitudinal changes in hearing and visual impairments and risk of dementia in older adults in the United States. JAMA Netw Open 2022; 5: e2210734. 35511175 10.1001/jamanetworkopen.2022.10734 PMC9073563

[71] Kwan RYC , Kwan CW, Kor PPK, Chi I. Cognitive decline, sensory impairment, and the use of audio-visual aids by long-term care facility residents. BMC Geriatr 2022; 22: 216. 35296238 10.1186/s12877-022-02895-x PMC8928635

[72] Davidson JGS , Guthrie DM. Older adults with a combination of vision and hearing impairment experience higher rates of cognitive impairment, functional dependence, and worse outcomes across a set of quality indicators. J Aging Health 2019; 31: 85–108. 28805100 10.1177/0898264317723407

[73] Utoomprurkporn N , Stott J, Costafreda SG, North C, Heatley M, Bamiou DE. The screening accuracy of a visually based Montreal cognitive assessment tool for older adult hearing aid users. Front Aging Neurosci 2021; 13: 706282. 34475818 10.3389/fnagi.2021.706282 PMC8406998

[74] Bainbridge KE , Wallhagen MI. Hearing loss in an aging American population: extent, impact, and management. Annu Rev Public Health 2014; 35: 139–52. 24641557 10.1146/annurev-publhealth-032013-182510

[75] Reischies FM , Geiselmann B. Age-related cognitive decline and vision impairment affecting the detection of dementia syndrome in old age. The British journal of psychiatry: The Journal of Mental Science 1997; 171: 449–51. 9463604 10.1192/bjp.171.5.449

[76] Phillips NA , Chertkow H, Pichora-Fuller MK, Wittich W. Special issues on using the Montreal cognitive assessment for telemedicine assessment during COVID-19. J Am Geriatr Soc 2020; 68: 942–4. 10.1111/jgs.16469 32253754

[77] Iliadou V , Moschopoulos N, Sidiras C, Eleftheriadou A, Nimatoudis I. Over-diagnosis of cognitive deficits in psychiatric patients may be the result of not controlling for hearing sensitivity and auditory processing. Psychiatry Clin Neurosci 2018; 72: 742. 29999211 10.1111/pcn.12768

