# Supplementary material for: Strategies used during the cognitive evaluation of older adults with dual sensory impairment: a scoping review
Source: Age Ageing. 2024 Mar 20;53(3):afae051. doi: 10.1093/ageing/afae051 (PMC10953621; doi:10.1093/ageing/afae051)
Supplement: aa-23-1214-File002_afae051 [file aa-23-1214-file002_afae051.docx]

**Strategies Used during the Cognitive Evaluation of Older Adults with Dual Sensory Impairment: A Scoping Review**

## **Appendix A:** *Example of search strategy for the MEDLINE database*

| **Concept** | **#** | **Searches** | **Results** |
| --- | --- | --- | --- |
|  | 1 | exp Neuropsychological Tests/ | 182118 |
|  | 2 | ((neuropsychologic* or neuro-psychologic* or Behavioral or Psychiatric or neuropsychiatric* or neuro-psychiatric* or memory or Bender-Gestalt or Bender Visual Motor Gestalt Test or language or Hooper Visual Organization or Controlled Oral Word Association or Continuous Performance or clock or Symbol Digit Modalities or vocabulary or Boston Naming or Paced Auditory Serial Addition or "Tower of London" or aphasia or developmental or Learning or Mental Navigation or stroop or trail making or Halstead Category or Seashore Rhythm or Speech Sounds Perception or Tactual Performance or Wisconsin Card Sorting) adj2 (test* or assess* or exam* or task* or status* or battery or interview* or scal*)).tw. | 144477 |
|  | 3 | ((Cognitive* or mental or dementia or neurocogniti* or neuro-cogniti* or alzheimer) adj (screening or Function? or test* or impair* or status* or scal* or decline? or assess*)).tw. | 159162 |
|  | 4 | (AX-CPT or CPT or NEPSY or CANTAB or TOMAL or FCRS or M-WCST or WCST or MMSE or MOCA or CPS or GPCOG or UPDRS Panel? or COGNISTAT or CDR or ADRDA).tw. | 45123 |
|  | 5 | (Delis-Kaplan Executive Function System or "Test of Everyday Attention" or Rey-Osterrieth Complex Figure? or stroop effect? or stroop paradigm? or Halstead-Reitan Battery or "Test of Memory Malingering" or "Alzheimer's Disease and Related Disorders Association").tw. | 2359 |
|  | 6 | ((Wechsler Memory or Wittenborn or Factor Construct Rating or Edinburgh Postnatal Depression or Katz Adjustment?) adj1 scal*).tw. | 4051 |
|  | 7 | ("Assessment of Cognition" or Mini Mental State Examination? or Unified Parkinson's Disease Rating Scale or Mini-Cog or MiniCog or Dementia Rating? or MicroCog or Micro-Cog).tw. | 20589 |
| **A** | 8 | 1 or 2 or 3 or 4 or 5 or 6 or 7 | 430716 |
|  | 9 | exp Deaf-Blind Disorders/ | 1123 |
|  | 10 | ((sensory or sensation) adj (impair* or loss* or disorder? or deficienc* or dysfunction? or defect? or handicap*) adj10 (dual or double or multi or multiple)).tw. | 320 |
|  | 11 | (Sensation Disorders/ or (exp Hearing Disorders/ and exp Vision Disorders/)) and (dual or double or multi or multiple).tw. | 948 |
|  | 12 | (((vision or visual*) adj (impair* or loss* or disorder? or deficienc* or dysfunction? or defect? or handicap*) adj10 (dual or double or multi or multiple)) and ((hearing or auditory or auditive) adj (impair* or loss* or disorder? or deficienc* or dysfunction? or defect? or handicap*) adj10 (dual or double or multi or multiple))).tw. | 49 |
|  | 13 | (Deaf* and blindness* and (dual or double or multi or multiple)).tw. | 124 |
|  | 14 | (dual impairment? or dual dysfunction? or "hearing and vision loss*" or "Vision and Hearing Loss*" or deafblind* or deaf-blind* or Deafness Blindness or Blind-Deaf* or Deaf-Mutism-Blind or "hearing and visual impairment?" or "hearing and visually impaired" or "hearing and visual disability" or "hearing and visual disabilities" or "hearing and visually disabled" or "vision and hearing impairment?" or "visually and hearing disabled" or "vision and hearing disability" or "vision and hearing disabilities" or "vision and hearing dysfunction" or "vision and hearing defect").tw. | 1124 |
|  | 15 | (((Usher or Hallgren or Wolfram) adj Syndrome?) or Dystrophia Retinae Pigmentosa-Dysostosis Syndrome? or Retinitis Pigmentosa Deafness Syndrome? or Deafness-Retinitis Pigmentosa Syndrome? or (Retinitis Pigmentosa and Congenital Deafness*)).tw. | 1701 |
| **B** | 16 | 9 or 10 or 11 or 12 or 13 or 14 or 15 | 3855 |
|  | 17 | exp aged/ or Housing for the Elderly/ or Homes for the Aged/ or Senior Centers/ or Adult Day Care Centers/ or Healthy Aging/ | 3239641 |
|  | 18 | (aged or old or older or elder* or frail or ageing or aging or senescence or "over 65" or "over 80" or "65 year*" or "85 year*" or Nonagenarian? or Octogenarian? or Centenarian? or ((retirement or senior?) adj (center? or centre? or home?))).tw. | 2315414 |
|  | 19 | Health Services for the Aged/ or Geriatrics/ or geriatric assessment/ or Geriatric Psychiatry/ or Geriatric Nursing/ or Geriatric Dentistry/ | 87523 |
|  | 20 | (geriatr* or psychogeriatr* or sociogeriatr* or Geronto* or Beers Criteria).tw. | 60344 |
|  | 21 | ((elder* or aged) adj2 (care or caring or healthcare or (Health adj (Service? or centre? or center? or facilt* or institution?)) or hospital or clinic? or institutionali#ed)).tw. | 13650 |
| **C** | 22 | 17 or 18 or 19 or 20 or 21 | 4898433 |
| **A and B and C** | 23 | 8 and 16 and 22 | 175 |
| **A and B and C limited** | 24 | limit 23 to (English or French or German or Spanish) | 168 |

## **Appendix B:** *Characteristics of sources of evidence*

| **#** | **Study** | **Total sample gender (men/women)** | **Participants’ mean age in years (SD) [range]** | **Characteristics of the sensory impairment** | **Vision measures used** | **Hearing measures used** | **Cognitive evaluation tool(s) used** | **Strategies used to modify test administration for sensory impairment** |
| --- | --- | --- | --- | --- | --- | --- | --- | --- |
| **1** | [1] | 250/635 | 81.0 (5.2) | - Hearing: > 5/12 word errors on the Whisper Test.  - Vision: > 50% using the near-vision Rosenbaum card. | - Near-vision Rosenbaum card | - Whisper test | Mini Mental State Examination (screening tool) | - |
| **2** | [2] | 39%/61% | 77.6 (7.6) | - Self-reported vision and hearing loss. | - Self-report | - Self-report | - Neuropsychological test battery (diagnostic tool) - Clinical Dementia Rating (diagnostic tool) | - |
| **3** | [3] | M_females_=0.639 (1=female, 0=male) | 78.171 (5.96) | Vision and hearing: Participants were classified as severely impaired, moderately impaired, and not impaired based on their self-report. | - Self-report | - Self-report | Two self-assessed measures of cognitive function (trouble remembering and frequency of confusion; screening tool) | - |
| **4** | [4] | 872/938 | [70-79] | - Vision – Visual acuity: 20/40 or worse or log contrast units 1.55 or less.  - Hearing – Pure tone average (PTA): Moderate-to-severe hearing loss corresponds to a PTA greater than 40 dB. | - Bailey–Lovie distance visual acuity test  - Pelli–Robson contrast sensitivity test | - Pure tone average | Mini Mental State Examination  (screening tool) | - |
| **5** | [5] | 19/41 | - With DSI: 81.5 (8.3) [63-92]  - With Dementia: 79.7 (5.5) [71-88]  - Controls: 77.66 (6.5) [69-96] | - Vision – etiologies: age-related macular degeneration (n = 10), diabetic retinopathy (n = 3), glaucoma (n = 3), cataract (n = 2), stroke (n = 2), retinitis pigmentosa (n = 1), Usher syndrome type 2 (n = 1), genetic other than Usher syndrome (n = 1), and unspecified (n = 2).  - Hearing – etiologies: presbycusis (n = 12), noise exposure (n = 5), genetic (other than Usher syndrome) (n = 4), Usher syndrome type 2 (n = 1), infection (n = 1), stroke (n = 1), and unspecified (n = 1). | - Not reported | - Not reported | Tactile test battery (screening tool) | (A) **The assistance of team members from related fields** (deafblind consultants) (C) **Communication strategies** (instructions given in tactile sign language, spoken language, by use of written texts or other communicative means)  (E) **Tests that do not include visual/audio items** (tactile test) |
| **6** | [6] | - No impairment: 444/487  - Single sensory impairment: 1 282/1 673  - Dual sensory impairment: 1 079/ 1 549 | - No impairment: 68.3 (6.1)  - Single sensory impairment: 68.8 (6.2)  - Dual sensory impairment: 72.6 (7.4)  - Total sample: [58–101] | - Self-evaluated hearing function. | - Self-report | - Self-report | Korean version of the Consortium to Establish a Registry for Alzheimer’s Disease (diagnostic tool) | (A) **The assistance of team members from related fields** (geriatric psychiatrist, clinical psychologist, and a nurse) |
| **7** | [7] | 68/49 | 41 (13.5) | Vision and hearing impairment etiologies: Rubella (n = 35), Down syndrome (n = 10), premature birth (n = 9), CMV (n = 5), CHARGE syndrome (n = 4), perinatal asphyxia (n = 4), Usher syndrome (n = 4), unknown (n = 22), other (n = 24). | - Medical case records  - Self report | - Medical case records  - Self report | Informant Questionnaire on Cognitive Decline in the Elderly (screening tool) | (A) **The assistance of team members from related fields** (deafblind consultant) |
| **8** | [8] | 36.8%/63.2% | ≥ 65 | - Vision: The vision item is scored from 0 to 4. Zero coincides with adequate vision, 1 represents mild vision impairment indicated by problems seeing regular size print (e.g., newspaper), 2 indicates moderate impairment represented by an inability to see large print (e.g., newspaper headlines) but able to see objects, 3 is highly impaired indicated by a problem identifying objects but the client was able to follow objects, and a 4 is associated with severe impairment (i.e., no vision).  - Hearing: The hearing item ranges from 0 to 3, with 0 representing adequate hearing, 1 indicates mildly impaired (e.g., difficulties when not in a quiet setting), 2 equals moderately impaired (e.g., speaker has to adjust quality/tone of speech), and 3 represents severely impaired (e.g., absence of useful hearing). | - Resident Assessment Instrument-Home Care  - Observation | - Resident Assessment Instrument-Home Care  - Observation | Cognitive Performance Scale (screening tool) | (A) **The assistance of team members from related fields** (Trained professionals) |
| **9** | [9] | - VI: 120/296  - HI: 310/218  - DSI: 117-122  - No impairment: 954/1371 | - VI: 71.04 (7.03)  - HI: 69.94 (6.48)  - DSI: 71.10 (7.22)  - No impairment: 68.2 (6.14) | - Vision: Reported difficulties in far (8.27%), near (10.13%), general vision (13.65%).  - Hearing: Reported difficulties in following a conversation (37.51%) and general hearing (21.87%). | - Self-report | - Self-report  - Observation | Four cognitive sub-tests (unnamed; screening tool) | (C) **Communication strategies** (Participants were assessed with their visual and hearing aids if they had them.) |
| **10** | [10] | - With dementia: 36.3%/63.6%  - Without dementia: 42.1%/57.9% | - With dementia: 81.9 (7)  - Without dementia: 76.4 (7) | - Vision: Reported difficulties.  - Hearing: Reported difficulties. | - Self-report | - Self-report | - Diagnosis (diagnostic tool) - ICD-9 dementia diagnosis codes | - |
| **11** | [11] | 36%/64% | 71.13 (7.4) | - Vision: Individuals with scores worse than 20/40 on Snellen tests of near or far visual were considered to have vision loss.  - Hearing: Individuals with pure-tone hearing thresholds of ≥ 26 dB were considered to have hearing loss. | - Snellen far visual acuity | - Audiometric evaluation (Words-in-noise test) | Montreal Cognitive Assessment-Blind (screening tool) | (B) **Modified scoring procedures** (eliminating visual items from the MoCA) |
| **12** | [12] | 60 154 590/67 764 016 | 61.31 [45-97] | - Vision and hearing: Self-reported difficulties. | - Self-report | - Self-report | American Community Survey | - |
| **13** | [13] | 141/154 | 81.46 (5.56) [73-100] | - Vision: Individuals with scores worse than 20/40 on Snellen test of binocular vision (corrected) were considered to have vision loss.  - Hearing: Individuals were considered to have hearing loss if they could not hear 25 dB at any frequency of a pure-tone hearing thresholds test. | - Snellen chart | - Audiometric evaluation (Pure tone thresholds test) | Telephone Interview for Cognitive Status (screening tool) | - |
| **14** | [14] | 37.5%/62.5 | ≥ 55 | - Vision: VI was defined as best corrected visual acuity of the better eye less than 39 Early Treatment Diabetic Retinopathy Study letters score.  - Hearing: Bilateral hearing impairment was determined as the pure-tone average of audiometric hearing thresholds at 500,1000, 2000, and 4000 Hz (PTA0.5-4kHz) in the better ear, defining any hearing loss as PTA0.5-4kHz > 25 dB HL; mild hearing loss as PTA0.5-4kHz > 25-40 dB HL; and moderate to severe hearing loss as PTA0.5-4kHz >40 dB HL. An audiologist asked questions about history of any self-perceived hearing problem, and if a hearing aid had been provided. Specifically, participants were asked: ‘Do you or have you ever worn a  hearing aid?’ (Yes/No/Don’t know). | - Early Treatment Diabetic Retinopathy Study chart  - LogMAR chart | - Pure-tone audiometry  - Self-report  - Hearing Handicap Inventory for the Elderly – Shortened version | Mini Mental State Examination (screening tool) | - |
| **15** | [15] | Not reported | ≥ 55 | - Vision: VI was defined as presenting or best-corrected visual acuity less than 20/40 (better eye).  - Hearing: HI was defined as average pure-tone air conduction threshold >25 dB HL (500–4,000 Hz, better ear). | Visual acuity | -Pure- tone average | Mini Mental State Examination (screening tool) | - |
| **16** | [16] | Not specified | [70-99] | - Vision: Vision impairment was defined as a score worse than 20/40 on visual acuity.  - Hearing: Self-report. | - Electronic Visual Acuity test  - Pelli-Robson chart  - Medical records | - Self-report | - Mini Mental State Examination (screening tool) - Trails B (paper and pencil test; screening tool) - Visual Closure Subtest of the Motor Free Visual Perception Test (screening tool) | - |
| **17** | [17] | 19.4%/80.6 | 84.3 | - Vision: VI was categorized as mild (reads large letters but not normal type in newspapers and books), moderate (cannot read newspaper headlines, but recognizes objects), severe (probably unable to recognize objects, but eyes can follow moving objects to no vision or can only see light, colors, or contours).  - Hearing: HI was categorized as mild (required quiet surroundings to hear  well), moderate (a person talking must speak loudly, clearly and precisely) and severe (extremely reduced hearing to no  hearing). | - Kombinert Alvorlig Sansesvikt  - Resident Assessment Instrument Acute Care | - Kombinert Alvorlig Sansesvikt  - Resident Assessment Instrument Acute Care | Cognitive Performance Scale (screening tool) | - |
| **18** | [18] | - Home care clients with DSI: 34.2%/65.8%  - Long-term care clients with DSI: 29.9%/69.9% | ≥ 65 | - DSI: The Deafblind Severity Index combines the functional hearing and vision items described above, to create a five-point scale (0 for no impairment in either sense to 5 for severe impairment in both senses). A score of three or higher on the DbSI was used to identify individuals with DSI since it represents the presence of at least mild impairment in both vision and hearing. | - Deafblind Severity Index from the Resident Assessment Instrument-Home Care  - Resident Assessment Instrument–Minimum Data Set 2.0 | - Deafblind Severity Index from the Resident Assessment Instrument-Home Care  - Resident Assessment Instrument–Minimum Data Set 2.0 | Cognitive Performance Scale (screening tool) | (A) **The assistance of team members from related fields** (registered nurses) |
| **19** | [19] | Not specified | Not specified | - Vision: The vision item captures the person’s ability to see close objects in adequate light while using their typical assistive device (e.g., reading glasses, magnifying glass), if required. It is scored on a five-point scale where 0 = adequate, 1 = impaired, 2 = moderately impaired, 3 = highly impaired and 4 = severely impaired.  - Hearing: The hearing item asks the assessor to rate the person’s hearing, with an appliance in place (e.g., hearing aid) if that is typically used, during the previous three days. It is scored on a 4-point scale where 0 = hears adequately, 1 = minimal difficulty, 2 = hears in special situations only and 3 = highly impaired.  - DSI: The Deafblind Severity Index combines these two items to create a scale that ranges from 0 (no  impairment in either sense) to 5 (severe impairment in both). | -Deafblind Severity Index from the Resident Assessment Instrument-Home Care | - Observation | Cognitive Performance Scale (screening tool) | (A) **The assistance of team members from related fields** (registered nurses and social workers) |
| **20** | [20] | 75 435/143 415 | 81.70 (7.5) | - DSI: DSI was determined using the Deafblind Severity Index, from two items in the RAI-HC, one each on functional hearing and vision, and ranges from 0 (no impairment in either sense) to 5 (severe impairment in both). | - Deafblind Severity Index from the Resident Assessment Instrument-Home Care | - Deafblind Severity Index from the Resident Assessment Instrument-Home Care | Cognitive Performance Scale (screening tool) | (A) **The assistance of team members from related fields** (registered nurses) |
| **21** | [21] | 107/122 | >60 | - Vision: A better eye acuity of ≤0.3 LogMar.  - Hearing: Categorized as (1) normal hearing level 4FA ≤25 decibels in hearing level, (2) mild hearing loss 4FA 26 to 40 decibels in hearing level, (3) moderate hearing loss 4FA 41 to 70 decibels in hearing level, or (4) severe hearing loss 4FA >70 decibels in hearing level. | - The lighthouse international chart  - Tumbling E (for participants unable to read or write) | - Otoscopic examination  - Screening tympanometry  - Air and bone conduction hearing thresholds  - Pure-tone hearing thresholds | Mini Mental State Examination (screening tool) | - |
| **22** | [22] | - VI: 41.32%/58.68%  - HI: 58.62%/41.38%  - DSI: 60.47%/49.33  - No Impairment: 50.67%/39.53% | 83.37 (4.51) | Not reported. | - Distance visual acuity chart  - Self-report | - Audiometric assessment of both ears  - Self-report | Four cognitive sub-tests (1. Counting Backwards from 100 for 30 seconds; 2. Digit Span Backwards subtest of the German version of the revised Wechsler Adult Intelligence Scale WAIS–R; 3. Animal Naming; 4. subtest Similarities taken from the German WAIS–R; screening tools) | (C) **Communication strategies** (speak slowly, clearly, and loudly, ask for feedback during conversation)  (D) **Environmental modifications** (testing took place in very quiet room)  (E) **Tests that do not include visual/audio items** (tests administered verbally only) |
| **23** | [23] | - No impairment: 764/544  - VI: 100/52  - HI: 159/171  - DSI: 53/40 | - No impairment: 66.8 (7.4)  - VI: 74.3 (8.4)  - HI: 73.4 (7.8)  - DSI: 80.4 (7.0) | -Vision: Impairment was defined as best-corrected visual acuity <6/12 in the worse-eye.  - Hearing: Hearing loss was defined as the pure-tone average of the audiometric hearing thresholds at 0.5, 1, 2 and 4 kilohertz (kHz) (PTA0.5–4 kHz) >40 decibels in the worse-ear. | - LogMAR chart  - Distance visual acuity chart  - Pinhole acuity  - Early Treatment Diabetic Retinopathy Study | - Pure-tone audiometry | Mini Mental State Examination-Blind (screening tool) | (B) **Modified scoring procedures** (excluding vision-related items of the MMSE) |
| **24** | [24] | - No impairment: 827/653  - Single impairment: 249/218  - DSI: 70/34 | - No impairment: 78.4 (3.1)  - Single impairment: 78.5 (3.2)  - DSI: 79.1 (3.5) | - Vision: Self-reported negative visual function.  - Hearing: Self-reported negative hearing function. | - Self-report | - Self-report | - Mini Mental State Examination (screening tool) - Clinical Dementia Rating (diagnostic tool) - Modified Mini-Mental State Examination (screening tool) - Cognitive subscale of the Alzheimer's Disease Assessment Scale (screening tool) - Neuropsychological battery of tests (diagnostic tool) | (A) **The assistance of team members from related fields** (adjudication committee for dementia classification) |
| **25** | [25] | 160/416 | 78.4 (6.9) [56-102] | - Vision: Visual impairment was defined with a Snellen of 20/70.  - Hearing: Hearing function was classified as impaired or intact. | - Lighthouse Near Visual Acuity Test | - Whisper test | Mini Mental State Examination (screening tool) | - |
| **26** | [26] | 274/350 | - Women: 69 (7.7) [57-90]  - Men: 68.8 (7.6) [57-92] | - Not reported. | - Early Treatment Diabetic Retinopathy Study Chart  - Groningen Edge Contrast cart  - Friedman Visual Field Analyzer  - Ability to read text | - Pure-tone air conduction audiometry | - Mini Mental State Examination (screening tool) - Cognitive subtest (short-term memory test derived from the verbal learning test; screening tools) | - |
| **27** | [27] | 636/616 | 78 [65-103] | - Vision: Visual loss was defined by corrected distance (3m) visual acuity >0.3  logMAR (equivalent to 20/40 or 6/12 vision) in the better eye, self-reported blindness, or glaucoma.  - Hearing: Hearing loss was defined as a pure-tone average >25 decibel in the better ear. | - Visual acuity  - Self-report | - Pure-tone audiometry | Mini Mental State Examination (screening tool) | - |
| **28** | [28] | 728/665 | 75.1 (1.5) [72-79] | - Vision: A visual acuity >0.3 logMAR was considered as poor.  - Hearing: Hearing described as an inconvenience. | - Visual acuity | - Self-report | Mini Mental State Examination (screening tool) | - |
| **29** | [29] | - No impairment: 41.7%/58.3%  - VI: 30.19%/69.81%  - HI: 54.13%/45.87%  - DSI: 40.01%/59.99% | >65 | - Vision: Individuals were considered to have functional vision impairment if they reported blindness, an inability to see well enough to recognize someone across the street, or inability to see well enough to read newspaper print.  - Hearing: Individuals were considered to have functional hearing impairment if they reported deafness, hearing aid use, inability to hear well enough to use the telephone, or inability to hear well enough to carry on conversation in a room with the television or radio playing. | - Self-report | - Self-report | - Diagnosis (diagnostic tool) - Self-reported diagnosis information - Measured cognitive performance in 3 domains (unnamed; screening tool) | - |
| **30** | [30] | 58/164 | 86.2 (7.4) | - Vision: Individuals were considered to have vision impairment if they scored less than the equivalent of 20/50 visual acuity.  - Hearing: Individuals were considered to have hearing impairment if their pure tone average was worse than or equal to 40 dBLs in both ears. | - Early Treatment of Diabetic Retinopathy Scale | - Pure tone audiometry | Mini Mental State Examination (screening tool) | - |
| **31** | [31] | 20 306/1 319 | 31.3 (8.6) [18-65] | - Vision: Self-reported vision problem.  - Hearing: Self-reported hearing difficulty. | - Self-report | - Self-report | 22-item Neurobehavioral Symptom Inventory (screening tool) | - |
| **32** | [32] | 2100/2521 | 64.9 (8.3) | - Vision: Self-reported vision.  - Hearing: Self-reported hearing. | - Self-report | - Self-report | Modified version of the cognitive score developed by Batty, Deary, and Zaninotto (2016) referring to working memory and executive function (screening tool) | - |
| **33** | [33] | 0/6112 | 76.1 | - Vision: Individuals were considered to have visual impairment if they had corrected binocular vision worse than 20/40.  - Hearing: Individuals were considered to have hearing impairment by their inability to hear a tone of 40 dB or greater at 2,000 Hz frequency in the better ear. | - Binocular visual acuity  - Bailey Lovie Targets | - Hand-held audiometer | Modified version of the Mini Mental State Evaluation (screening tool) | - |
| **34** | [34] | 1664/2167 | - Men: 72.3 (5.86)  - Women:73.9 (6.62) | - Vision: Self-reported vision.  - Hearing: Self-reported hearing. | - Self-report | - Self-report | Mini Mental State Examination (screening tool) | - |
| **35** | [35] | 6373/6724 | 58.5 (9.3) | - Vision: Self-reported distance vision.  - Hearing: Self-reported hearing. | - Self-report | - Self-report | Validated instruments (unnamed) | (A) **The assistance of team members from related fields** (trained interviewers) |
| **36** | [36] | 90/120 | 72.46 (5.52) | - Vision: Individuals were considered to have visual impairment if they had habitual visual acuity in the better eye of >0.3 LogMAR.  - Hearing: Individuals were considered to have hearing impairment if they had a hearing threshold of >25 decibels hearing level at 4 frequency average (0.5, 1, 2 and 4 kHz) in the better ear. | - Early Treatment Diabetic Retinopathy Study | - Pure-tone audiometry  - Air-conduction threshold | Montreal Cognitive Assessment (screening tool) | - |
| **37** | [37] | - Sample 1: 41.8%/58.2%  - Sample 2: 45.6 %/54.4%  - Sample 3: 45.6%/54.4% | - Sample 1: 67.8 (3.4)  - Sample 2: 64.8 (10)  - Sample 3: 64.8 (10.1) | - Vision: Self-reported vision quality.  - Hearing: Self-reported hearing function. | - Self-report | - Self-report | Three surveys (unnamed) | - |
| **38** | [38] | - Total sample: 10,871/8747  - No impairment: 8,310/6119  - HI: 468/1080  - VI: 1,619/1080  - DSI: 474/510 | 57.8 (6.2) | - Vision: Self-reported vision quality.  - Hearing: Self-reported hearing function. | - Self-report | - Self-report | - Telephone Interview for Cognitive Status (screening tool)  - Three cognitive sub-tests (episodic memory, serial 7s, backward counting; screening tools) | (E) **Tests that do not include visual/audio items** (The TICS is administered over the phone and does not include visually presented items). |
| **39** | [39] | 605/1149 | - Men: 80.89 (7.39)  - Women: 82.45 (6.95) | Not reported. | - Observation | - Observation | Functional assessment (unnamed) | (A) **The assistance of team members from related fields** (trained and certified investigators) |
| **40** | [40] | - No impairment: 38.5%/61.5%  - VI: 46.9%/53.1%  - HI: 47.6 %/52.4%  - DSI: 52.4%/47.6% | - No impairment.: 68.17 (4.94) [59.7–80.3]  - VI: 69.13 (5.30) [60.1–78.9]  - HI: 72.20 (5.61) [60.0–80.3]  - DSI: 73.59 (4.60) [64.2–80.8] | - Vision: Vision impairment was defined as a visual function mean deviation on visual field testing worse than -5 decibels in the better eye.  - Hearing: Hearing impairment was defined as four-frequency (0.5±4 kHz) pure tone average (PTA) threshold worse than 25 dB in  the better ear. | - Visual field examination (Zeiss Humphrey Field Analyzer II 750i) | - Bilateral otoscopy  - Pure tone air-conduction audiometry | Mini Mental State Examination- Blind (screening tool) | (B) **Modified scoring procedures** (The MMblind omits 8 visually presented items) |
| **41** | [41] | 1148/2349 | 79.8 (3.9) | - Vision: Self-reported vision performance.  - Hearing: Self-reported hearing performance. | - Self-report | - Self-report | - Mini Mental State Examination (screening tool) - Structured interview for the diagnosis of dementia of the Alzheimer type (diagnostic tool) - Global Deterioration Scale (screening tool) - Blessed Dementia Rating Scale (screening tool) - Clinical Dementia Rating (diagnostic tool) | (A) **The assistance of team members from related fields** (research assistants, psychologists, geriatricians, or geriatric psychiatrists) |
| **42** | [42] | 541/842 | 74.4 (9.4) | - Vision: Individuals were considered to have low visual acuity if they had habitual visual acuity worse than 20/40 in the better eye or >0.3 LogMAR.  - Hearing: Individuals were considered to have low hearing acuity if they had a hearing threshold of >25 decibels hearing level at 4 frequency average (500, 1,000, 2,000 and 4,000 Hz.) in the better ear. | - Bailey-Lovie chart  - Self-report | - Welch Allyn portable audiometer  - Pure-tone auditory threshold | - Mini Mental State Examination (screening tool) - Trail-making Test - Part B of the Halsted Reitan Battery (screening tool)  - Verbal fluency test (screening tool) | - |
| **43** | [43] | 55/158 | 86.38 (7.32) | - Vision impairment was categorized as mild (best ETDRS in both eyes 33-35 letters); moderate (best ETDRS in both eyes 8-32 letters); and legally blind (best ETDRS in both eyes ≤ 7 letters).  - Hearing impairment was categorized based on pure tone average levels in the best ear as moderate (41-55 dBL), moderately severe (56-70 dBL) and severe (greater than 71 dBLs). | - Early Treatment of Diabetic Retinopathy Study (ETDRS) | - Audiometry (Madson 304 portable audiometer) | Mini Mental State Examination (screening tool) | (A) **The assistance of team members from related fields** (research nurses) |
| **44** | [44] | 98/124 | >60 | - Vision: Individuals were considered to have visual impairment if the visual acuity was  less than 6/18 in the better eye with the best available correction. Those with visual acuity less than 6/60 in the better eye with best available correction were considered blind.  - Hearing: Hearing impairment was categorized based on pure tone average levels in the best ear as “no impairment” at or below 25 dB, 26–40 dB “mild impairment”, 41–55 dB “moderate impairment” and more than 55 dB as “severe impairment”. | - Snellen’s E chart | - Pure tone audiometry | Mini Mental State Examination (screening tool) | - |
| **45** | [45] | 8794/9244 | 59.9 (9.7) | - Vision: Self-reported vision function.  - Hearing: Self-reported hearing function. | - Self-report | - Self-report | - Telephone Interview for Cognitive Status (screening tool)  - Two cognitive subtests (word recall test, pentagon drawing test; screening tools) | (E) **Tests that do not include visual/audio items** (The TICS is administered over the phone and does not include visually presented items). |
| **46** | [46] | 1021/1397 | 68.6 [43–84] | - Vision: Mild or greater VI was defined as  best-corrected visual acuity of 20/40 or worse in the better eye.  - Hearing: Mild or greater HI was defined as a pure-tone average of the air conduction thresholds at 0.5, 1, 2, and 4 kHz greater than 25 dB hearing Level in either ear. | - Early Treatment of Diabetic Retinopathy Study | - Pure-tone air and bone conduction audiometry | Mini Mental State Examination (screening tool) | - |
| **47** | [47] | 587/1061 | 82.6 (6.2) | - Vision: A patient was considered to have low vision when visual acuity in the  better eye was < 20/60. Mild vision loss was defined as visual acuity < 20/40, but ≥ 20/60. Near vision was abnormal when near visual acuity was worse than Parinaud 2 in at least one eye. Amsler grid testing was considered abnormal when patients described scotoma and/or metamorphopsia.  - Hearing: Not reported. | -Visual acuity measurement (Snellen and Parinaud charts)  - Amsler grid | - Hearing Handicap Inventory for the Elderly Screening | Mini Mental State Examination (screening tool) | - |
| **48** | [48] | - No impairment: 283/189  - HI: 49/80  - VI: 51/33  - DSI: 29/26 | - No impairment: 72.09 (5.67)  - HI: 75.78 (6.35)  - VI: 73.87 (5.95)  - DSI: 73.89 (6.55) | - Vision: Self-reported vision function.  - Hearing: Self-reported hearing function. | - Self-report | - Self-report | Blessed Orientation-Memory-Concentration test (screening tool) | - |
| **49** | [49] | 39%/61% | 81.3 | - Vision: Vision loss was characterized by a visual acuity in the better eye with best standard correction of 20/60 ft (6/18 m) or  less, or a visual field diameter of < 60 degrees in the better eye, or hemianopsia (loss of half of the visual field).  - Hearing: Hearing loss was characterized by an unaided pure-tone average decibel hearing loss (dB HL) in the better ear of 35 dB  HL or more across 4 frequencies: 0.2, 1, 2, and 4 kHz. | - Medical records | - Medical records | - Montreal Cognitive Assessment-Blind (screening tool) - Cognitive Performance Scale (screening tool) | (B) **Modified scoring procedures** (eliminating auditory and visual items from the MoCA) |
| **50** | [50] | - VI: 41.32%/58.68%  - HI: 58.62%/41.38%  - DSI: 60.47%/39.53%  - No impairment: 50.67%/49.33% | - VI: 82.60 (4.63)  - HI: 82.69 (5.08)  - DSI: 83.37 (4.51)  - No impairment: 82.6 (4.5) | - Vision: Best-corrected distance and/or near visual acuity of participants in the VI sample also had to be no more than .30 in the better eye (approximately 20/70 in the U.S. metric).  - Hearing: Regarding the hearing-impaired sample, the average hearing loss  in decibels (dB HL) at frequencies of 500, 1000, and 2000 Hz had to be at least 35 dB HL in the better ear. | Not reported | Not reported | Four cognitive sub-tests (1. counting backwards from 100 for 30; 2. digit-span backwards subtest of the German version of the revised Wechsler Adult Intelligence scale (WAIS-R); 3. animal naming; 4. similarities subtest of the German WAIS-R (screening tools) | (A) **The assistance of team members from related fields** (trained research assistant with psychology background) (C) **Communication strategies** (speak slowly, clearly, and loudly, ask for feedback during conversation, encourage participants to wear hearing aids if applicable and available) (D) **Environmental modifications** (testing took place in very quiet room) |
| **51** | [51] | 37.5%/62.5% | 82.5 (7.9) | - Vision: VI was measured using a single item within the RAI-HC that ranged from zero (no impairment) to four (severely impaired).  - Hearing: The presence of HI was identified by a single item within the Resident Assessment Instrument-Home Care that scores perceived functional hearing ability from zero (no impairment) to three (highly impaired).  - DSI: A score of three or higher on the Deafblind Severity Index identified clients with a DSI of both vision and hearing. | - Resident Assessment Instrument-Home Care | - Resident Assessment Instrument-Home Care | Cognitive Performance Scale (screening tool) | - |
| **52** | [52] | 4938/5082 | 58.9 (10.4) | -Vision: VI was defined as visual acuity <20/40 but >20/200 in the better seeing eye, and blindness was defined as VA <20/200 in the better-seeing eye. Based on the World Health Organization definition, VI was defined as VA <20/60 but >20/400 in the better-seeing eye, and blindness was defined as VA <20/400 in the better-seeing eye. The authors used a modified definition to classify individuals with VA of counting fingers or worse as blind.  - Hearing: Deafness was defined based on self-reported history of hearing loss. | - Visual acuity  - Subjective refraction  - Slit lamp examination  - Intraocular pressure measurement  - dilated fundus examination  - Fundus photography | - Self-report | Abbreviated Mental Test (screening tool) | - |
| **53** | [53] | 28.2%/71.8% | 83.3 | - Vision: The vision item was coded as 0 (adequate) if being able to see fine details, including regular print in newspapers with glasses or with other visual appliance normally used; 1 (minimum impairment) if able to see large print but not regular print in newspapers; 2 (moderate impairment) if not able to see newspaper headlines but can identify objects; 3 (severe impairment) if object identification is in questions but eyes appear to follow objects; 4 (no vision presents).  - Hearing: The hearing item was coded as 0 (adequate), suggesting no difficulty in normal conversation with hearing appliance normally used; 1 (minimum impairment) suggesting difficulty in some environments; 2 (moderate impairment) suggesting problem with hearing normal conversation, requiring quiet setting to hear well; 3 (severe impairment) suggesting difficulty in all situations; and 4 (cannot hear anything). | - InterRAI (Resident Assessment Instrument) Long-Term Care Facilities | - InterRAI (Resident Assessment Instrument) Long-Term Care Facilities | Cognitive Performance Scale (screening tool) | (A) **The assistance of team members from related fields** (study researchers) |
| **54** | [54] | 26.9%/73.1% | 83.4 (9.4) | - Vision: Vision impairment was defined in this study for residents experiencing minimal difficulty seeing with glasses or other visual appliance normally used (seeing large print, but not regular print in newspapers/books) to no vision at all.  - Hearing: Hearing impairment was defined as experiencing minimal difficulty (difficulty hearing in some environments, eg, when a person speaks softly and is more than 6 feet away), with hearing aid normally applied to no hearing at all. | - InterRAI (Resident Assessment Instrument) Long-Term Care Facilities | - InterRAI (Resident Assessment Instrument) Long-Term Care Facilities | Cognitive Performance Scale (screening tool) | (A) **The assistance of team members from related fields** (study researchers, nursing home staff) |
| **55** | [55] | 1294/1093 | 77.5 (2.8) | - Vision: An acuity equivalent of 20/50 or worse in the better-presenting eye was defined as impaired. ≤1.3 log units of contrast sensitivity was defined as impaired.  - Hearing: A pure-tone average >25 decibels hearing level is defined as any hearing impairment. | -Bailey-Lovie distance visual acuity test  - Pelli-Robson contrast sensitivity | - Audiometric assessment  - Pure-tone air conduction thresholds | Mini Mental State Examination (screening tool) | - |
| **56** | [56] | 4113/3011 | ≥ 65 | - Vision: Vision impairment was defined as self-reported blindness, or not being able to see across the street and/or newspaper print (despite using glasses or contacts if  applicable).  - Hearing: Hearing impairment was defined as any of the following: self-reported deafness, using a hearing aid or other hearing device, or not being able to hear well enough to use the telephone or to carry on a conversation in a room with a radio or TV playing (using a hearing device if applicable). | - Self-report | - Self-report | - Self or proxy reported physician diagnosis (diagnostic tool)  - AD8 Dementia Screening Interview (screening tool)  - Unnamed cognitive test (screening tool) | - |
| **57** | [57] | 201/530 | 88.1 (2.7) [85-98] | - Vision: Moderate to severe vision impairment was defined as a best corrected visual acuity of <6/18 but ≥3/60 in the better eye or binocularly, and blindness as a BCVA of <3/60 in the better eye or binocularly.  - Hearing: Hearing impairment was categorized as mild hearing impairment (hearing loss score of 11–17), moderate hearing impairment (score of 8–24), moderately severe hearing impairment (score of 25–31), severe hearing impairment (score of 32–38), and profound hearing  impairment (score of 39–44). | - Best-corrected visual acuity | - Hearing Handicap Inventory for the Elderly Screening Version  - Self-report question | Mini Mental State Examination (screening tool) | - |
| **58** | [58] | 5363/6359 | 83.7 (11.1) | - Vision: Self-reported vision function.  - Hearing: Self-reported hearing function. | -Self-report | - Self-report | Mini Mental State Examination (screening tool) | - |
| **59** | [59] | 6297/4379 | ≥65 | - Vision: Self-reported vision function.  - Hearing: Self-reported hearing function. | - Self report | - Self-report | - Self-reported physician diagnosis (diagnostic tool)  - AD8 Dementia Screening Interview (screening tool)  - Unnamed cognitive test (screening tool) | - |
| **60** | [60] | At least 60% of the sample participants were female | ≥65 | - Vision: VI was defined as a score of one or higher on a single item that scores vision from zero (no impairment) to four (severely impaired).  - Hearing: The presence of HI was defined as a score of one or higher on a single item on the RAI-HC. This item scores hearing ability from zero (no impairment) to three (highly impaired).  - DSI was defined as a score of three or higher on the Deafblind Severity Index. | - Self-report - Proxy report  - Medical records | - Self-report - Proxy report  - Medical records | Cognitive Performance Scale (screening tool) | (A) **The assistance of team members from related fields** (trained care coordinators, registered nurses) |
| **61** | [61] | 49.1%/50.9% | 63 (10.25) [45-85] | - Vision: Self-reported vision function. Participants with visual acuity > 0.2  logMAR (<20/32 or 6/10) were categorized as having VI that was at least mild.  - Hearing: Self-reported hearing function. participants with a > 25 dB HL pure tone audiometry of the better ear were characterized as having a hearing impairment. | - Self-report  - Visual Acuity | - Self-report  - Audiometric thresholds | Cognitive tests (Mental Alternation Test, Animal Fluency Test, Controlled Oral Word Association Test, and the Stroop test, Rey Auditory Verbal Learning Test; screening tools) | - |
| **62** | [62] | 88/202 | 81.1 (12.7) [50-100] | - Vision: Self-reported vision function.  - Hearing: Self-reported hearing function. | - Self-report | - Self report | Reported diagnosis (diagnostic tool) | - |
| **63** | [63] | 1223/1704 | 74.6 (4.8) | - Vision: Self-reported vision function.  - Hearing: Self-reported hearing function. | - Self-report | - Self-report | Mini Mental State Examination (screening tool) | (A) **The assistance of team members from related fields** (trained clinical study staff) |
| **64** | [64] | 703/1530 | 82.1 (8.2) | - Vision: Participants’ visual impairment was classified as either “No Impairment” referred to seeing fine details adequately, including regular or large print in newspapers or books, “Impairment” (e.g., not able to see newspaper  headlines but able to identify objects) or “Severe impairment” (e.g., no vision or only able to see light, colours, or shapes.  - Hearing: Hearing impairment was classified as “No Impairment” described as being able to adequately hear normal talk, TV, and phone, “Impairment” (e.g., having difficulty hearing when not in a quiet setting) and “Highly impaired” (e.g., absence of  useful hearing). | - Vision Patterns section of the Minimum Data Set-Resident Assessment Instrument Version 2.0 | - Hearing Patterns section of the Minimum Data Set-Resident Assessment Instrument Version 2.0 | Cognitive Performance Scale (screening tool) | (A) **The assistance of team members from related fields** (nurses, social workers, occupational therapists, and physiotherapists) |
| **65** | [65] | 1331/1599 | 73.5 (6.1) | - Vision: Self-reported vision function.  - Hearing: Self-reported hearing function. | - Self-report | - Self-report | Mini Mental State Examination (screening tool) | - |
| **66** | [66] | - Delirium: 288/488  - No delirium: 884/1376 | - Delirium: 84.7 (7.1)  - No delirium: 82.2 (7.6) | - Vision: Visual impairment was diagnosed when a patient was unable to see the fingers of the interviewer at 1 meter of distance during daylight with or without aids.  - Hearing: Hearing impairment was diagnosed when a patient was unable to understand the interviewer’s words at a normal voice volume at distance of 1 meter with or without aids. | - Observation | - Observation | - Diagnosis (diagnostic tool)  - Medication prescription | - |
| **67** | [67] | 6692/7222 | - No sensory impairment: 58.12 (9.60)  - VI: 62.66 (10.12)  - HI: 66.45 (11.29)  - DSI: 67.70 (11.40) | - Vision: Self-reported vision problems.  - Hearing: Self-reported hearing problems. | - Self-report | - Self-report | Telephone Interview for Cognitive Status (screening tool) | - |

**Appendix C:** *Bibliography of included sources of evidence*

1. Agostini JV, Han L, Tinetti ME. The Relationship Between Number of Medications and Weight Loss or Impaired Balance in Older Adults. *Journal of the American Geriatrics Society* 2004; **52**: 1719–23.

2. Bhojak T, Jia Y, Jacobsen E *et al.* Driving Habits of Older Adults: A Population-based Study. *Alzheimer disease and associated disorders* 2021; **35**:2 50–7.

3. Brennan M, Horowitz A, Su Y. Dual Sensory Loss and Its Impact on Everyday Competence. *The Gerontologist* 2005; **45**: 337–46.

4. Brenowitz WD, Kaup AR, Lin FR *et al.* Multiple Sensory Impairment Is Associated With Increased Risk of Dementia Among Black and White Older Adults. *The journals of gerontology Series A, Biological sciences and medical sciences* 2019; **74**: 890–6.

5. Bruhn P, Dammeyer J. Assessment of Dementia in Individuals with Dual Sensory Loss: Application of a Tactile Test Battery. *Dementia and geriatric cognitive disorders extra* 2018; **8**: 12–22.

6. Byeon G, Oh GH, Jhoo JH *et al.* Dual Sensory Impairment and Cognitive Impairment in the Korean Longitudinal Elderly Cohort. *Neurology* 2021; **96**: e2284–95.

7. Dammeyer J. Interaction of Dual Sensory Loss, Cognitive Function, and Communication in People who are Congenially Deaf-Blind. *Journal of Visual Impairment & Blindness* 2010; **104**: 719–25.

8. Davidson JGS, Guthrie DM. Older Adults With a Combination of Vision and Hearing Impairment Experience Higher Rates of Cognitive Impairment, Functional Dependence, and Worse Outcomes Across a Set of Quality Indicators. *Journal of Aging and Health* 2019; **31**: 85–108.

9. de la Fuente J, Hjelmborg J, Wod M *et al.* Longitudinal Associations of Sensory and Cognitive Functioning: A Structural Equation Modeling Approach. *The journals of gerontology Series B, Psychological sciences and social sciences* 2019; **74**: 1308–16.

10. Deardorff WJ, Liu PL, Sloane R *et al.* Association of Sensory and Cognitive Impairment with Healthcare Utilization and Cost in Older Adults. *Journal of the American Geriatrics Society* 2019; **67**: 1617–24.

11. Dupuis K, Pichora-Fuller MK, Chasteen AL *et al.* Effects of hearing and vision impairments on the Montreal Cognitive Assessment. *Neuropsychology, development, and cognition Section B, Aging, neuropsychology and cognition* 2015; **22**: 413–37.

12. Fuller SD, Mudie LI, Siordia C *et al.* Nationwide Prevalence of Self-Reported Serious Sensory Impairments and Their Associations with Self-Reported Cognitive and Functional Difficulties. *Ophthalmology* 2018; **125**: 476–85.

13. Ge S, McConnell ES, Wu B *et al.* Longitudinal Association Between Hearing Loss, Vision Loss, Dual Sensory Loss, and Cognitive Decline. *Journal of the American Geriatrics Society* 2021; **69**: 644–50.

14. Gopinath B, Liew G, Burlutsky G *et al.* Association between vision and hearing impairment and successful aging over five years. *Maturitas* 2021; **143**: 203–8.

15. Gopinath B, Mcmahon CM, Burlutsky G *et al.* Hearing and vision impairment and the 5-year incidence of falls in older adults. *Age and ageing* 2016; **45**: 409–14.

16. Green KA, Jr GM, Owsley C. Associations between Visual, Hearing, and Dual Sensory Impairments and History of Motor Vehicle Collision Involvement by Older Drivers. *Journal of the American Geriatrics Society* 2013; **61**: 252–7.

17. Grue EV, Kirkevold M, Ranhoff AH. Prevalence of vision, hearing, and combined vision and hearing impairments in patients with hip fractures. *Journal of Clinical Nursing* 2009; **18**: 3037–49.

18. Guthrie DM, Davidson JGS, Williams N *et al.* Combined impairments in vision, hearing and cognition are associated with greater levels of functional and communication difficulties than cognitive impairment alone: Analysis of interRAI data for home care and long-term care recipients in Ontario. *PloS one* 2018; **13**: e0192971.

19. Guthrie DM, Declercq A, Finne-Soveri H *et al.* The Health and Well-Being of Older Adults with Dual Sensory Impairment (DSI) in Four Countries. *PloS one* 2016; **11**: e0155073.

20. Guthrie DM, Thériault ÉR, Davidson JGS. Self-Rated Health, Cognition, and Dual Sensory Impairment Are Important Predictors of Depression Among Home Care Clients in Ontario. *Home Health Care Management & Practice* 2016; **28**: 35–43.

21. Harithasan D, Mukari SZS, Ishak WS *et al.* The impact of sensory impairment on cognitive performance, quality of life, depression, and loneliness in older adults. *International journal of geriatric psychiatry* 2020; **35**: 358–64.

22. Heyl V, Wahl H-W. Managing Daily Life With Age-Related Sensory Loss: Cognitive Resources Gain in Importance. *Psychology and aging* 2012; **27**: 510–21.

23. Hong T, Mitchell P, Burlutsky G *et al.* Visual Impairment, Hearing Loss and Cognitive Function in an Older Population: Longitudinal Findings from the Blue Mountains Eye Study. *PloS one* 2016; **11**: e0147646.

24. Hwang PH, Jr WTL, Thielke SM *et al.* Dual sensory impairment in older adults and risk of dementia from the GEM Study. *Alzheimer’s & dementia (Amsterdam, Netherlands)* 2020; **12**: e12054.

25. Keller BK, Morton JL, Thomas VS *et al.* The Effect of Visual and Hearing Impairments on Functional Status. *Journal of the American Geriatrics Society* 1999; **47**: 1319–25.

26. Kempen GI, Verbrugge LM, Merrill SS *et al.* The impact of multiple impairments on disability in community-dwelling older people. *Age and ageing* 1998; **27**: 595–604.

27. Kiely KM, Anstey KJ, Luszcz MA. Dual sensory loss and depressive symptoms: the importance of hearing, daily functioning, and activity engagement. *Frontiers in Human Neuroscience* 2013; **7**: 1–13.

28. Kiely KM, Mortby ME, Anstey KJ. Differential associations between sensory loss and neuropsychiatric symptoms in adults with and without a neurocognitive disorder. *International psychogeriatrics* 2018; **30**: 261–72.

29. Kuo P-L, Huang AR, Ehrlich JR *et al.* Prevalence of Concurrent Functional Vision and Hearing Impairment and Association With Dementia in Community-Dwelling Medicare Beneficiaries. *JAMA network open* 2021; **4**: e211558.

30. Lach HW, Lozano AJ, Hanlon AL *et al.* Fear of Falling in Sensory Impaired Nursing Home Residents. *Aging & mental health* 2020; **24**: 474–80.

31. Lew HL, Pogoda TK, Baker E *et al.* Prevalence of Dual Sensory Impairment and Its Association With Traumatic Brain Injury and Blast Exposure in OEF/OIF Veterans. *The Journal of head trauma rehabilitation* 2011; **26**: 489–96.

32. Liljas AEM, Walters K, de Oliveira C *et al.* Self-Reported Sensory Impairments and Changes in Cognitive Performance: A Longitudinal 6-Year Follow-Up Study of English Community-Dwelling Adults Aged ⩾50 Years. *Journal of aging and health* 2020; **32**: 243–51.

33. Lin MY, Gutierrez PR, Stone KL *et al.* Vision Impairment and Combined Vision and Hearing Impairment Predict Cognitive and Functional Decline in Older Women. *Journal of the American Geriatrics Society* 2004;**52**:1 996–2002.

34. Lyu J, Kim H-Y. Gender-Specific Associations of Sensory Impairments with Depression and Cognitive Impairment in Later Life. *Psychiatry investigation* **15**: 926–34.

35. Ma X, Wei J, Congdon N *et al.* Longitudinal Association Between Self-Reported Sensory Impairments and Episodic Memory among Older Adults in China: A Prospective Cohort Study. *Journal of geriatric psychiatry and neurology* 2021; **35**: 382–91.

36. Mah HY, Ishak WS, Rahman MHA. Prevalence and risk factors of dual sensory impairment among community‐dwelling older adults in Selangor: A secondary data analysis. *Geriatrics & gerontology international* 2020;**20**: 911–16.

37. Maharani A, Dawes P, Nazroo J *et al.* Visual and hearing impairments are associated with cognitive decline in older people. *Visual and hearing impairments are associated with cognitive decline in older people* 2018; **47**:5 75–81.

38. Maharani A, Dawes P, Nazroo J *et al.* Associations Between Self-Reported Sensory Impairment and Risk of Cognitive Decline and Impairment in the Health and Retirement Study Cohort. *The journals of gerontology Series B, Psychological sciences and social sciences* 2020; **75**: 1230–42.

39. Mitoku K, Masaki N, Ogata Y *et al.* Vision and hearing impairments, cognitive impairment and mortality among long-term care recipients: a population-based cohort study. *BMC geriatrics* 2016; **16**: 1–7.

40. Mudie LI, Varadaraj V, Gajwani P *et al.* Dual sensory impairment: The association between glaucomatous vision loss and hearing impairment and function. *PloS one* 2018; **13**: e0199889.

41. Pabst A, Bär J, Röhr S *et al.* Do self‐reported hearing and visual impairments predict longitudinal dementia in older adults? *Journal of the American Geriatrics Society* 2021; **69**: 1519–28.

42. Parada H, Laughlin GA, Yang M *et al.* Dual impairments in visual and hearing acuity and age-related cognitive decline in older adults from the Rancho Bernardo Study of Healthy Aging. *Age and ageing* **50**: 1268–76.

43. Petrovsky DV, Sefcik JS, Hanlon AL *et al.* Social Engagement, Cognition, Depression and Co-morbidity in Sensory Impaired Nursing Home Residents. *Research in gerontological nursing* 2019; **12**: 217–26.

44. R. D, Kasthuri A. Visual and hearing impairment among rural elderly of south India: A community‐based study. *Geriatrics & gerontology international* 2012; **12**: 116–22.

45. Rong H, Lai X, Jing R *et al.* Association of Sensory Impairments With Cognitive Decline and Depression Among Older Adults in China. *JAMA network open* 2020; **3**: e2014186.

46. Schubert CR, Fischer ME, Pinto AA *et al.* Sensory Impairments and Risk of Mortality in Older Adults. *The journals of gerontology Series A, Biological sciences and medical sciences* 2017; **72**: 710–5.

47. Soler V, Sourdet S, Balardy L *et al.* Visual Impairment Screening at the Geriatric Frailty Clinic for Assessment of Frailty and Prevention of Disability at the Gérontopôle. *The journal of nutrition, health & aging* 2016; **20**: 870–7.

48. Soto-Perez-de-Celis E, Sun C-L, Tew WP *et al.* Association between Patient-Reported Hearing and Visual Impairment and Functional, Psychological, and Cognitive Status among Older Adults with Cancer. *Cancer* 2018; **124**: 3249–56.

49. Alfaro AU, Guthrie DM, Phillips NA *et al.* Detection of vision and /or hearing loss using the interRAI Community Health Assessment aligns well with common behavioral vision/hearing measurements. *PloS one* 2019; **14**: e0223123.

50. Wahl H-W, Heyl V, Drapaniotis PM *et al.* Severe vision and hearing impairment and successful aging: a multidimensional view. *The Gerontologist* 2013; **53**: 950–62.

51. Williams N, Phillips NA, Wittich W *et al.* Hearing and Cognitive Impairments Increase the Risk of Long-term Care Admissions. *Innovation in Aging* 2020; **4**: igz053.

52. Wong TY, Tham Y-C, Sabanayagam C *et al.* Patterns and Risk Factor Profiles of Visual Loss in a Multiethnic Asian Population: The Singapore Epidemiology of Eye Diseases Study. *American journal of ophthalmology* 2019; **206**: 48–73.

53. Yamada Y, Denkinger MD, Onder G *et al.* Dual Sensory Impairment and Cognitive Decline: The Results From the Shelter Study. *The journals of gerontology Series A, Biological sciences and medical sciences* 2016; **71**: 117–23.

54. Yamada Y, Vlachova M, Richter T *et al.* Prevalence and Correlates of Hearing and Visual Impairments in European Nursing Homes: Results From the SHELTER Study. *Journal of the American Medical Directors Association* 2014; **15**: 738–43.

55. Armstrong NM, Vieira Ligo Teixeira C, Gendron C *et al.* Associations of dual sensory impairment with incident mobility and ADL difficulty. *Journal of the American Geriatrics Society* 2022; **70**: 1997–2007.

56. Assi L, Ehrlich JR, Zhou Y *et al.* Self-reported dual sensory impairment, dementia, and functional limitations in Medicare beneficiaries. *Journal of the American Geriatrics Society* 2021; **69**: 3557–67.

57. Bikbov MM, Kazakbaeva GM, Rakhimova EM *et al.* Concurrent vision and hearing impairment associated with cognitive dysfunction in a population aged 85+ years: the Ural Very Old Study. *BMJ Open* 2022; **12**: e058464.

58. Chen L, Zhou R. Association of sensory impairment with cognitive function and mental health among the older adults in China. *Current Psychology* 2022, DOI: 10.1007/s12144-022-02807-7.

59. Chen SP, Azad AD, Pershing S. Bidirectional Association between Visual Impairment and Dementia Among Older Adults in the United States Over Time. *Ophthalmology* 2021; **128**: 1276–83.

60. Guthrie DM, Williams N, Campos J *et al.* A Newly Identified Impairment in Both Vision and Hearing Increases the Risk of Deterioration in Both Communication and Cognitive Performance. *Canadian Journal on Aging / La Revue canadienne du vieillissement* 2022; **41**: 363–76.

61. Hämäläinen A, Pichora-Fuller MK, Wittich W *et al.* Self-report Measures of Hearing and Vision in Older Adults Participating in the Canadian Longitudinal Study of Aging are Explained by Behavioral Sensory Measures, Demographic, and Social Factors. *Ear and hearing* 2021; **42**: 814–31.

62. Hovaldt HB, Crowe K, Dammeyer J. A cross-sectional study of prevalence and correlates of self-harm and suicidal ideation in older adults with dual sensory loss. *Disability and Health Journal* 2022; **15**: 101204.

63. Hwang PH, Longstreth WT, Thielke SM *et al.* Longitudinal Changes in Hearing and Visual Impairments and Risk of Dementia in Older Adults in the United States. *JAMA Network Open* 2022; **5**: e2210734.

64. Kwan RYC, Kwan CW, Kor PPK *et al.* Cognitive decline, sensory impairment, and the use of audio-visual aids by long-term care facility residents. *BMC Geriatrics* 2022; **22**: 216.

65. Lee J, Suh Y, Park J *et al.* Combined effects of handgrip strength and sensory impairment on the prevalence of cognitive impairment among older adults in Korea. *Sci Rep* 2022; **12**: 6713.

66. Morandi A, Inzitari M, Udina C *et al.* Visual and Hearing Impairment Are Associated With Delirium in Hospitalized Patients: Results of a Multisite Prevalence Study. *Journal of the American Medical Directors Association* 2021; **22**: 1162-1167.e3.

67. Zhao X, Zhou Y, Wei K *et al.* Associations of sensory impairment and cognitive function in middle-aged and older Chinese population: The China Health and Retirement Longitudinal Study. *Journal of Global Health* 2021; **11**: 08008.
